# Genetic association analysis in sugarcane (*Saccharum* spp.) for sucrose accumulation in humid environments in Colombia

**DOI:** 10.1186/s12870-024-05233-y

**Published:** 2024-06-18

**Authors:** Carolina Saavedra-Díaz, Jhon Henry Trujillo-Montenegro, Hugo Arley Jaimes, Alejandra Londoño, Fredy Antonio Salazar Villareal, Luis Orlando López, Carlos Arturo Viveros Valens, Jershon López-Gerena, John J. Riascos, Yeison Mauricio Quevedo, Fernando S. Aguilar

**Affiliations:** 1 Centro de Investigación de la Caña de Azúcar de Colombia (CENICAÑA), Cali, Colombia; 2 Pontificia Universidad Javeriana, Cali, Colombia; 3 Colombian Sugarcane Research Center (Cenicaña), km 26 Vía Cali-Florida, Valle del Cauca, Colombia

**Keywords:** Sucrose, GWAS, Sugarcane, Humid environments, Sucrose

## Abstract

**Background:**

Sucrose accumulation in sugarcane is affected by several environmental and genetic factors, with plant moisture being of critical importance for its role in the synthesis and transport of sugars within the cane stalks, affecting the sucrose concentration. In general, rainfall and high soil humidity during the ripening stage promote plant growth, increasing the fresh weight and decreasing the sucrose yield in the humid region of Colombia. Therefore, this study aimed to identify markers associated with sucrose accumulation or production in the humid environment of Colombia through a genome-wide association study (GWAS).

**Results:**

Sucrose concentration measurements were taken in 220 genotypes from the Cenicaña’s diverse panel at 10 (early maturity) and 13 (normal maturity) months after planting. For early maturity data was collected during plant cane and first ratoon, while at normal maturity it was during plant cane, first, and second ratoon. A total of 137,890 SNPs were selected after sequencing the 220 genotypes through GBS, RADSeq, and whole-genome sequencing. After GWAS analysis, a total of 77 markers were significantly associated with sucrose concentration at both ages, but only 39 were close to candidate genes previously reported for sucrose accumulation and/or production. Among the candidate genes, 18 were highlighted because they were involved in sucrose hydrolysis (SUS6, CIN3, CINV1, CINV2), sugar transport (i.e., MST1, MST2, PLT5, SUT4, ERD6 like), phosphorylation processes (TPS genes), glycolysis (PFP-ALPHA, HXK3, PHI1), and transcription factors (ERF12, ERF112). Similarly, 64 genes were associated with glycosyltransferases, glycosidases, and hormones.

**Conclusions:**

These results provide new insights into the molecular mechanisms involved in sucrose accumulation in sugarcane and contribute with important genomic resources for future research in the humid environments of Colombia. Similarly, the markers identified will be validated for their potential application within Cenicaña’s breeding program to assist the development of breeding populations.

**Supplementary Information:**

The online version contains supplementary material available at 10.1186/s12870-024-05233-y.

## Background

Sugarcane (*Saccharum* spp.) is a major agronomic and industrial crop used to produce sugar, ethanol, and electricity worldwide and represents an important component of the economy of tropical and subtropical countries [1, 2]. Colombia ranks 14th among the world’s largest sugar producers and 12th among the largest exporters, accounting for 1.1% of the world’s sugar trade [3]. Given the economic importance of the crop, breeding programs around the world have directed their efforts in producing genotypes with high biomass (i.e., tons of cane per hectare or TCH), high sucrose content, and resistance to the most limiting diseases of the crop [4–6]. In general, this process involves making decisions based on the phenotypic information collected during the early stages of selection (three stages in most breeding programs) and multi environmental trials (METs), which provide information on genotype adaptability to the environment or to the target population of environments [4–7].

In Colombia, the sugarcane breeding process is carried out by the Colombian sugarcane research center, Cenicaña, which has been releasing varieties specifically adapted to the agroecological conditions of the Cauca River Valley since 1990. Recently, Cenicaña´s breeding program seeks to make its process more efficient by incorporating molecular markers. These markers have been used mainly with a genome wide association study (GWAS) and with a genomic prediction strategy trying to assist the selection of the best genotypes for complex traits like sucrose content, water stress, biomass production, among several others. In specific, GWAS have allowed an accurate and rapid identification of genes associated with traits of interest within sugarcane breeding programs around the world [5, 8]. For instance, using AFLP, DArT, and SSR technologies, six markers were found to be associated with the resistance to yellow leaf virus (SCYLV) in sugarcane [9] and six with the sugarcane brown rust resistance [10]. In Argentina, a total of 43 DArT markers were significantly associated with biomass (kg Plot^− 1^) and 38 with sugar content in a panel of 88 sugarcane clones [11]. These markers have also been used as part of a genomic selection strategy by incorporating them within the model training phase at the Estación Experimental Agroindustrial Obispo Colombres (EEAOC) Argentina [12]. Similarly, for stalk diameter, leaf width, leaf length, stalk number, internode length, brix, total weight, dry weight, and water content, a total of 217 SNP-type markers were significantly associated in a panel of 308 sugarcane clones in the USA [13]. They also found that from the 217 SNP-type markers, ten were involved in sugar metabolism, specifically with synthases, hydrolases, and transferases [13]. Finally, the marker G1 has been used as a molecular marker associated with the resistance to the sugarcane orange rust [14]. This marker had the ability to predict 65.8% of resistant phenotypes in the original mapping population [14] and 71.43% in a collection of resistant Brazilian cultivars [15] and therefore, it has been used within the breeding schemes in Brazil.

Sucrose content plays an important role in the genetic improvement of crops. This trait depends on various genetic factors, such as enzymes (i.e., sucrose synthase, sucrose phosphate synthase, and invertase), sucrose transporters, environmental factors, and biological processes (i.e., phosphorylation, hydrolysis, and regulatory mechanisms) [16]. For the accumulation of sucrose, the main enzymes reported are sucrose phosphate synthase (SPS), invertase, and sucrose synthase (SUS) [16]. SPS is involved in sucrose synthesis and accumulation, controlling the flux of carbon into sucrose and the movement of photosynthates from source to sink tissues [17–19]. The invertase enzymes generate a concentration gradient from source to sink by irreversibly hydrolyzing sucrose and producing an equimolar mixture of glucose and fructose necessary for cell elongation [17–20]. Sucrose synthase (SUS) is a glycosyltransferase enzyme involved in the reversible conversion of sucrose into fructose and UDP-glucose, both of which are required for respiration, starch biosynthesis, and fiber development [21, 22].

Sucrose accumulation in sugarcane is influenced by the crop cycle and variety planted [6]. For instance, it has been reported a difference between the plant cane and the ratoons, with the accumulation in the ratoons commonly being higher than the accumulation in the plant cane. However, even when the plant cane tends to have a lower sucrose (%Cane) than the ratoons, the difference in the varieties tend to remain stable. For instance, under the conditions of China the varieties with a high, intermediate, and low sucrose content have kept his rank throughout the plant cane, first, and second ratoon [6]. Similar behavior has been observed in Colombia where the curve of sucrose (% Cane) across plant cane and first ratoon have the same tendency between different varieties regardless of the crop cycle [23]. Additionally, when analyzing the commercial database for the 4 most planted varieties in the Cauca River Valley, Colombia, the sugar yield was stable across the crop cycles with 11.00 ± 0.62, 11.05 ± 0.61, and 11.04 ± 0.57 (%Cane) for plant cane, first, and second ratoon, respectively, suggesting that the differences between genotypes tend to be stable throughout the crop cycles even when the accumulation of sucrose was slightly higher in the first ratoon. This behavior could be attributed to the climatic conditions in Colombia. The agro-industrial sector of sugarcane in Colombia is located mainly in the Cauca River Valley, a region with a well-defined bimodal pattern with two rainy and two dry seasons [24], in which the harvest is carried out throughout the year without zafra conditions [25]. To improve the agronomic practices for the crop, in 2001, Cenicaña classified the fields of the region within 51 agroecological zones that integrate soil texture, climate, and water resources [26]. These agroecological zones have allowed the classification of the region within the semidry, humid, and foothill environments, which help to improve the biomass and sugar content for the varieties planted, reducing the difference between the crop cycles for biomass and sucrose content.

**Fig. 1:**
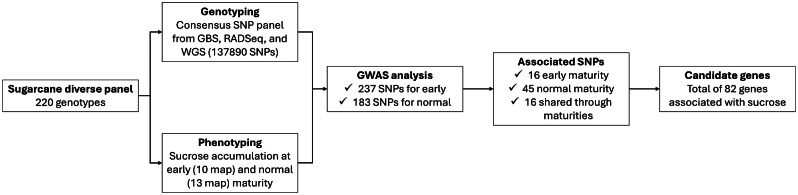
A graphical abstract of the methodology, analysis, and results presented in this study. The analysis of phenotypic and genotype data (consensus panel) is presented following a linear mixed model. The results of the association analysis (77 associated markers) are subsequently shown. Finally, the 82 genes identified as candidate genes are presented, whose function is involved in the accumulation and/or production of sucrose

Some studies have concluded that the accumulation of sucrose in sugarcane depends on different environmental factors, with soil moisture being the most important during the ripening process [27, 28]. During this process, a delay in plant growth is generated when a decrease in the soil and plant moisture is observed [6, 8, 28], as well as a decline in stalk fresh weight due to dehydration [29]. The sucrose content, expressed in percent cane (%Cane), is calculated based on fresh weight, which translates to a high variability linked to the soil moisture or daytime stomatal activity [30, 31]. Under a dry season, the decrease in internal humidity reduces sugar consumption for growth, and therefore, an increase in sucrose synthesis due to the conversion of reducing sugars into sucrose is produced [5, 28]. In contrast, during the rainy season or under high soil moisture, there is high vegetative growth and low accumulation of sucrose [32]. Brazil have reported an exponential relationship between the rainfall observed 120 days before harvesting and the total recoverable sugars, suggesting that when the precipitation is above 100 mm, a gradual decline in the total recoverable sugars is observed, mainly in late varieties [32]. In Colombia, a similar pattern was found with the southern zone of the Cauca River Valley, classified as a humid environment [25, 33]. Given that increasing the sucrose content in areas with high humidity can be a challenge due to the impact of environmental conditions on the plant’s physiology and sugar accumulation, this study aimed to identify molecular markers and candidate genes associated with sucrose accumulation and/or production in a diverse population in the humid environments of the Cauca River Valley, Colombia. A schematic representation of the analysis and results is presented in Fig. 1.

## Results

### Sequencing data

A total of 51.27, 458.74, and 7,012.48 GB data was obtained after sequencing the 220 genotypes from the diverse panel with GBS, RADSeq, and WGS, respectively. The GBS data (with an average depth of 105X) had 2.97 ± 1.49 million reads per sample on average, with a read length of 71.50 ± 1.40 base pairs. For the paired-end RADSeq (with an average depth of 27.0X) and WGS (with an average depth of 39X), there were 12.11 ± 2.69 and 108.00 ± 133.40 million reads per sample on average, respectively. Similarly, for RADSeq and WGS the read length was higher with 87.50 ± 3.10 bp and 138.00 ± 3.40 bp, respectively. After quality control, the reads from each sequencing technology were mapped to CC 01-1940 sugarcane reference genome [34] with an average mapping percentage of 71.30, 30.50, and 85.10 for GBS, RADSeq and WGS, respectively. Subsequently, the aligned data from each technology were merged within a consensus SNP panel, resulting in a total of 137,889 high-quality SNPs used for further analysis (Fig. 1).

### Phenotypic analysis

Based on Bonferroni-adjusted p-values, 3 and 24 data points were identified as outliers for early (10 months after planting) and normal maturity (13 months after planting), respectively (data not shown). Outliers are defined as data points that fall outside of the majority of the data for a particular subject and can mask the real distribution of the data [35, 36]. Because of this, outliers are commonly identified and removed from the analysis to reduce the impact on the estimation process of the traits of interest [35]. The accumulation of sucrose (%Cane) showed a continuous normal distribution with a mean of 12.64% at early maturity and 14.37% at normal maturity. For normal maturity, the best-fitting model includes all random effects, while for early maturity, a model excluding the genotype for crop cycle interaction was selected (Table 1). Similarly, for normal maturity, the model allows for heterogeneous residual variance across crop cycles ($V\left({\epsilon }_{ijkl}\right)={\sigma }_{e\left(i\right)}^{2}$), while for early maturity, the residual variance was homogeneous (Table 1). Broad sense heritability was 0.90 and 0.83 for early and normal maturity, respectively. The higher heritability values observed in this study are an indication of the high data quality and the good experimental design implemented, both of which help in reducing residual (${\sigma }_{e}^{2})$ variance. For early maturity, the genotypic effect contributed a significant proportion of the total variance with a value of 2.58, while for normal maturity, the residual variance had a larger effect with 2.94 (Table 2).

**Table 1:** Bayesian information criteria (BIC) values for each of the 16 possible models. All models contained the crop cycle as a fixed effect and genotype as a random effect, combined with the other random effects (denoted with an X)

Random effects				Heterogeneous residual variance	BIC	
*G*	*R*	*B*	*I*		Early	Normal
x	x	x	x	Yes	5314.8	4300.1
x	x	x	x	No	5312.5	4445.1
x		x	x	Yes	5330.1	4301
x		x	x	No	5327.4	4444.7
x	x		x	Yes	5330.1	4305.7
x	x		x	No	5330.5	4459.2
x			x	Yes	5401	4319.3
x			x	No	5395.7	4477.6
x	x	x		Yes	5310.6	4306.8
x	x	x		No	5308.4	4525.6
x		x		Yes	5326.1	4307.2
x		x		No	5323.5	4525
x	x			Yes	5328.3	4312
x	x			No	5325.4	4533.9
x				Yes	5401	4323.7
x				No	5395.7	4544.7

G = genotype, R = replication nested within the crop cycle, B = block nested within the replication and crop cycle, and *I* = genotype by crop cycle interaction

**Table 2:** Variance components for the accumulation of sucrose (%Cane) at early (10 months after planting) and normal (13 months after planting) maturity

Variance component	Residual variance	Variance	
		Early	Normal
$G$		2.58	1.90
$B$		0.10	0.03
$R$		0.14	0.05
$I$		-	0.50
Residual	Homogeneous	1.54	-
Residual (Plant Cane)	Heterogeneous	-	2.94
Residual (First ratoon)		-	0.68
Residual (Second ratoon)		-	1.35

G = genotype, R = replication nested within a crop cycle, B = block nested within the replication and crop cycle, and *I* = genotype by crop cycle interaction

### Population structure

**Fig. 2:**
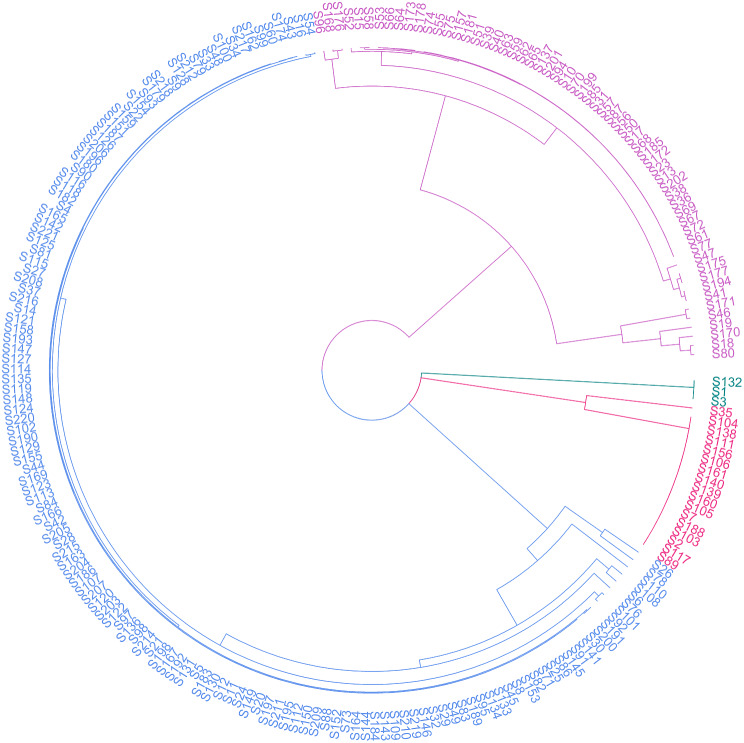
Population structure analysis of 220 sugarcane genotypes based on 137,889 SNPs. The purple and magenta branches correspond to subpopulations 1 and 2, respectively. The cyan branches refer to subpopulation 3, and the yellow branches refer to subpopulation 4. There are two main branches, the first for subpopulations 1 and 2 (purple and magenta), and the second includes subpopulations 3 and 4 (cyan and yellow). The image was created by the authors using the information deposited in data availability

The 220 genotypes were classified into four subpopulations (Fig. 2**).** There were six genotypes (S18, S46, S78, S80, S170, and S171) with a posterior probability indicating that they belong to two or more subpopulations. For these cases, the genotypes were assigned to the subpopulation to which they had the highest membership probability. The first and second subpopulations were mainly composed of hybrids, while the third grouped the genotypes from *S. spontaneum, S. officinarum, S. sinense, S. barberi*, and some interspecific genotypes (Fig. 2). The fourth subpopulation had only the genotypes S1, S132, and S3, all from the genus *Erianthus spp.* (Fig. 2).

### Association analysis and candidate genes

**Fig. 3:**
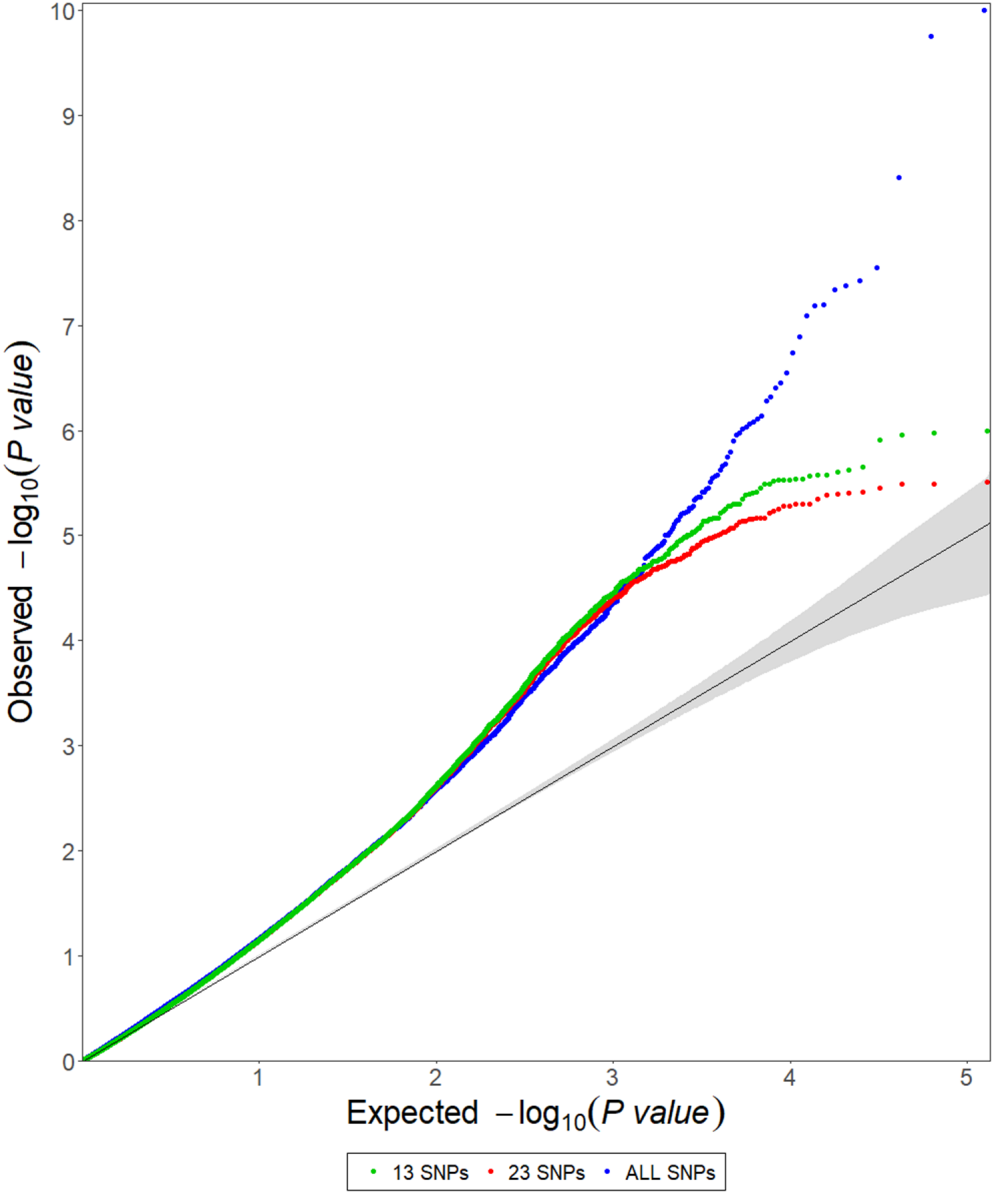
Quantile-quantile (QQ) plots for sucrose accumulation (%Cane) for the general model with sequential removal of markers based on score (-log10 p-value) for early maturity. The black lines represent the theoretical expected values, and the gray shaded regions represent the 95% confidence interval. The green points represent the model with all SNP markers

**Fig. 4:**
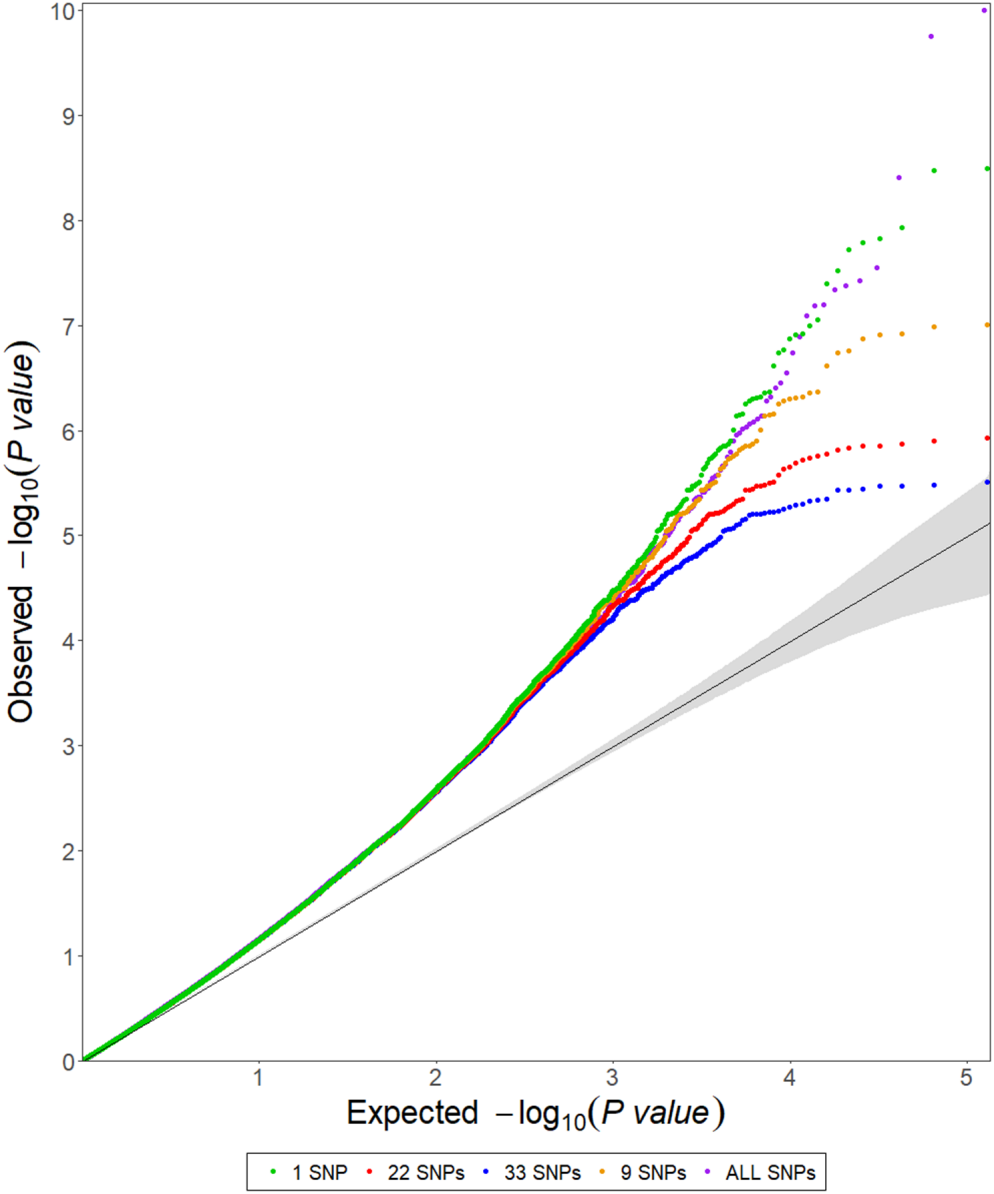
Quantile-quantile (QQ) plots for sucrose accumulation (%Cane) for the general model with sequential removal of markers based on score (-log10 p-value) for normal maturity. The black lines represent the theoretical expected values, and the gray shaded regions represent the 95% confidence interval. The green points represent the model with all SNP markers

**Fig. 5:**
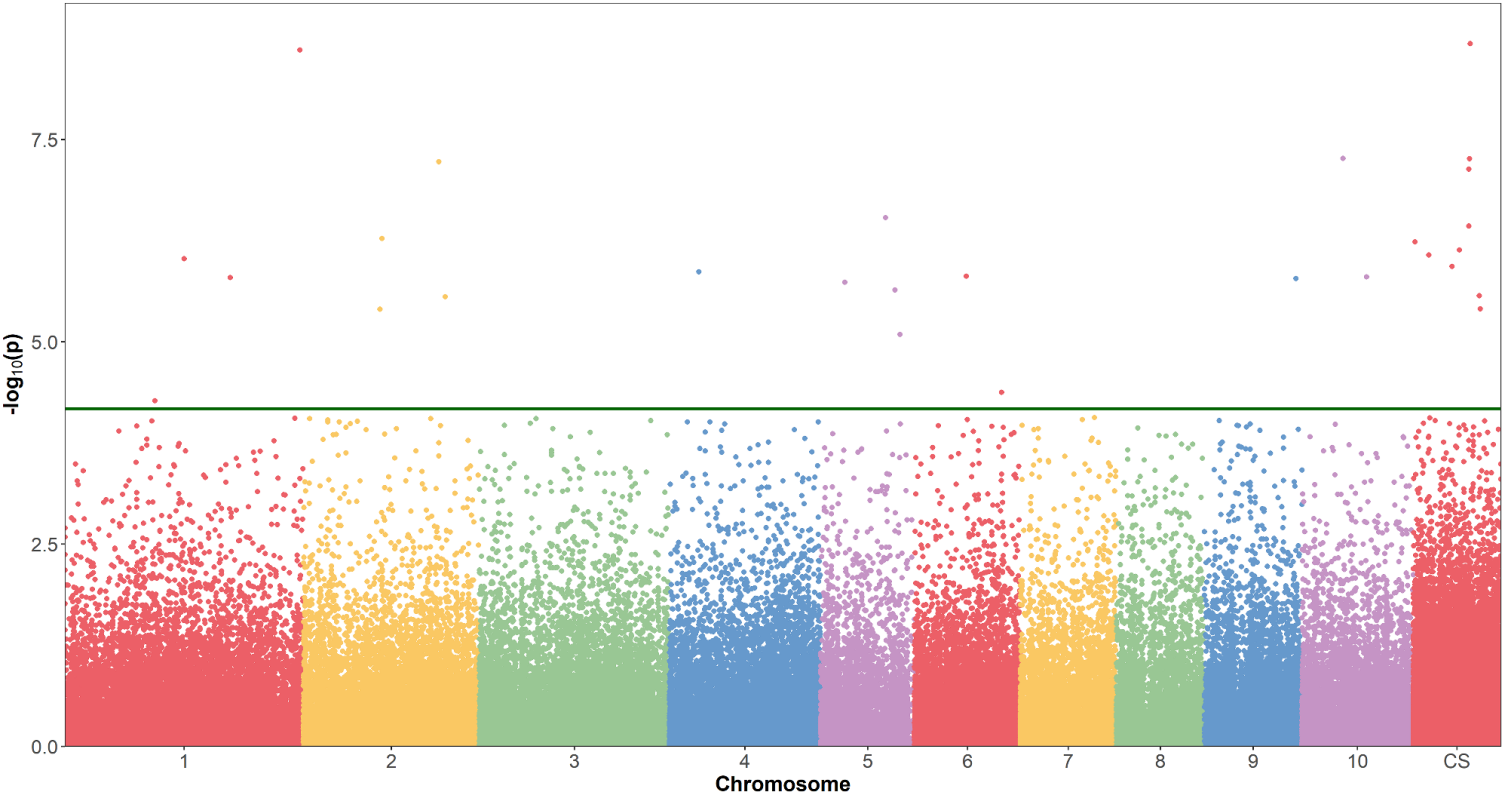
Manhattan plot for the general model, showing the significance of each SNPs (logarithmic scale) in the accumulation of sucrose at early maturity (10 months after planting) along the monoploid genome of the variety CC 01-1940 (chromosomes numbered 1 to 10, CS indicating contigs and scaffolds). The green line indicates the genome-wide threshold of *p* = 1 × 10-5. The score is presented on the Y axis (-log10 p-value)

**Fig. 6:**
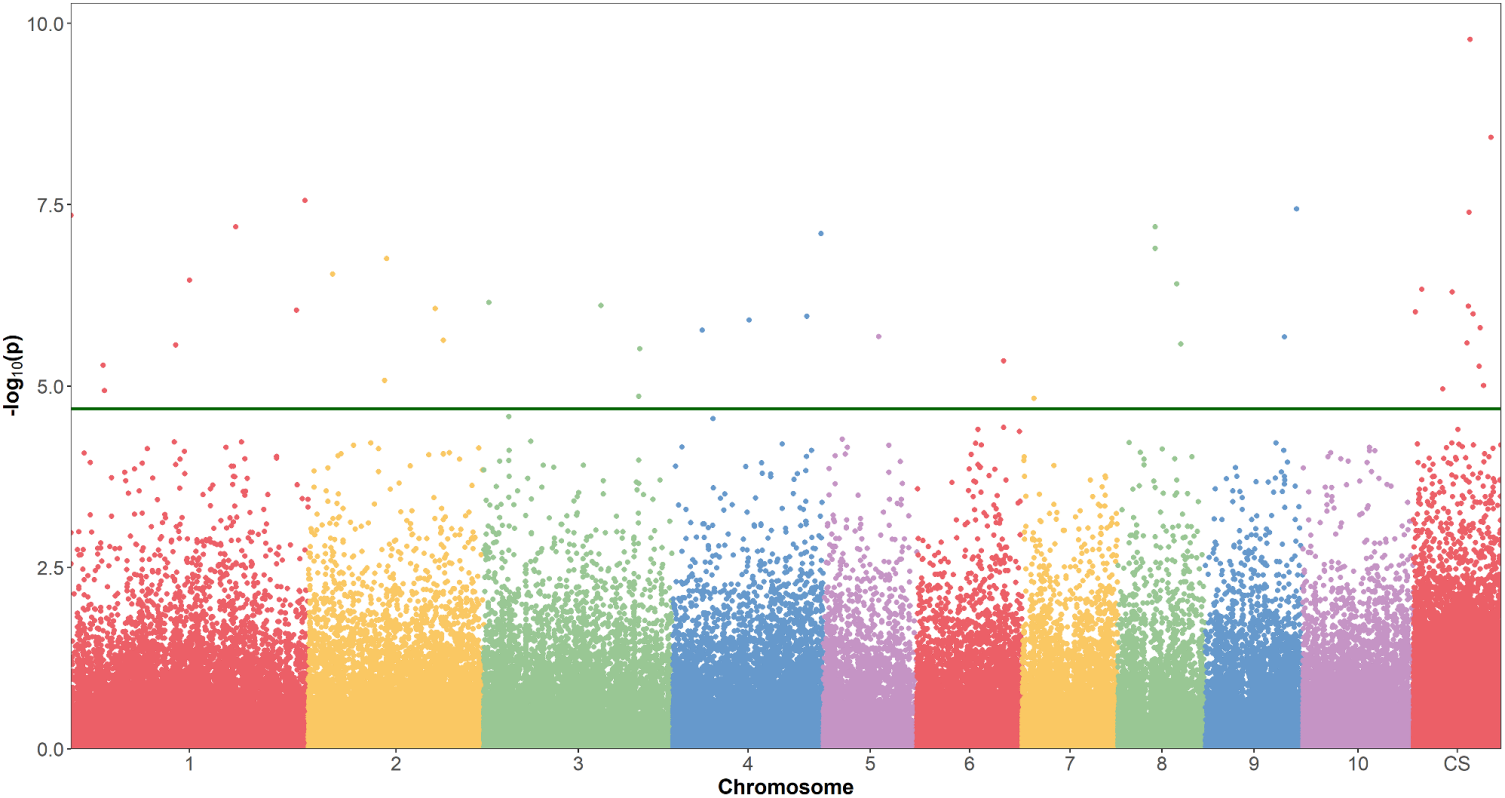
Manhattan plot for the general model, showing the significance of each SNPs (logarithmic scale) in the accumulation of sucrose at normal maturity (13 months after planting) along the monoploid genome of the variety CC 01-1940 (chromosomes numbered 1 to 10, CS indicating contigs and scaffolds). The green line indicates the genome-wide threshold of *p* = 1 × 10-5. The score is presented on the Y axis (-log10 p-value)

For early and normal maturity, there were 237 and 183 markers associated with sucrose (%Cane), respectively, with the general model having the highest number of markers for both maturities (Table 3). After removing the most significant markers and reanalyzing the general model, a total of 192 and 103 markers were identified as false positives for early (Fig. 3) and normal maturity (Fig. 4), respectively. There were 4 markers identified as false positives for the 2-dom-ref model at early maturity, while 11 markers were false positives for the 1-dom-ref model at normal maturity (Table 3). For the other genetic models, no false positives were identified (Table 3). After this filtering process, a total of 109 markers, 41 for early maturity and 69 for normal maturity, were retained for further analysis (Table 3, Additional file 2). The general model allowed the identification of the highest number of markers for both early (Fig. 5) and normal (Fig. 6) maturity. When analyzing the associated markers per genetic model, a total of 6 (1_33380771, 10_15141188, 4_10932960, contig_39315_31775, contig_50499_5072, and contig_65540_9617) and 8 (1_63454860, 3_1957031, 4_49815442, 4_55115204, 8_13879677, contig_39799_65, contig_40813_18644, and contig_50499_5072) were found associated between two or more genetic models for early and normal maturity, respectively. For these cases, the model in which the marker has the highest R^2^ was selected. Therefore, there were 77 markers, 16 for early, 45 for normal, and 16 shared between both maturities (Table 4). Finally, the highest percentage of phenotypic variation explained by each of the 77 markers (R^2^) was 25.39% (Table 4).

**Table 3:** Total number of associated markers (unfiltered) and markers identified as true associations (True positives) for the accumulation of sucrose (%Cane) at early (10 months after planting) and normal (13 months after planting) maturity

Model	Early		Normal	
	Unfiltered	True positives	Unfiltered	True positives
General	215	23	137	34
Additive	0	0	0	0
1-dom-alt	2	2	5	5
1-dom-ref	4	4	24	13
2-dom-alt	0	0	1	1
2-dom-ref	11	7	8	8
3-dom-alt	0	0	0	0
3-dom-ref	0	0	4	4
4-dom-alt	1	1	1	1
4-dom-ref	2	2	2	2
5-dom-alt	0	0	0	0
5-dom-ref	2	2	1	1
**Total**	**237**	**41**	**183**	**69**

**Table 4:** Marker effect and pseudo R^2^ per genetic model for the 77 SNPs significantly associated with sucrose accumulation at early (10 map) and normal (13 map) maturities

Trait	Model	Marker	Chromosome	Position (bp)	Reference allele	Alternative allele	Allele dosage	Marker effect	R2
Early	1-dom-alt	5_29476338	5	29,476,338	C	T		0.77	11.99
Early	1-dom-ref	1_33380771	1	33,380,771	A	C		-1.20	11.50
Early	1-dom-ref	4_55115204	4	55,115,204	C	G		1.37	14.02
Early	2-dom-ref	10_1273014	10	1,273,014	A	C		1.97	12.55
Early	2-dom-ref	2_28319736	2	28,319,736	C	T		-1.80	17.98
Early	2-dom-ref	3_20905930	3	20,905,930	A	T		-1.01	11.24
Early	2-dom-ref	contig_39799_65	contig_39799	65	C	T		2.02	15.78
Early	4-dom-alt	3_57539943	3	57,539,943	A	G		1.60	14.21
Early	5-dom-ref	6_32163815	6	32,163,815	A	C		1.64	11.83
Early	general	1_44113121	1	44,113,121	A	G	0	-1.09	20.81
Early	general	1_44113121	1	44,113,121	A	G	1	0.54	20.81
Early	general	1_44113121	1	44,113,121	A	G	2	-0.67	20.81
Early	general	1_44113121	1	44,113,121	A	G	3	-2.72	20.81
Early	general	1_44113121	1	44,113,121	A	G	4	-1.67	20.81
Early	general	1_44113121	1	44,113,121	A	G	5	2.38	20.81
Early	general	1_44113121	1	44,113,121	A	G	10	0.47	20.81
Early	general	1_61256982	1	61,256,982	C	T	0	-2.89	18.68
Early	general	1_61256982	1	61,256,982	C	T	1	-3.52	18.68
Early	general	1_61256982	1	61,256,982	C	T	2	-0.83	18.68
Early	general	1_61256982	1	61,256,982	C	T	3	-2.18	18.68
Early	general	1_61256982	1	61,256,982	C	T	10	-0.17	18.68
Early	general	1_86961880	1	86,961,880	A	G	0	-3.42	23.55
Early	general	1_86961880	1	86,961,880	A	G	1	-2.25	23.55
Early	general	1_86961880	1	86,961,880	A	G	2	-2.43	23.55
Early	general	1_86961880	1	86,961,880	A	G	3	-2.21	23.55
Early	general	1_86961880	1	86,961,880	A	G	10	0.04	23.55
Early	general	10_15141188	10	15,141,188	A	G	0	-5.28	18.09
Early	general	10_15141188	10	15,141,188	A	G	1	-1.01	18.09
Early	general	10_15141188	10	15,141,188	A	G	2	-0.21	18.09
Early	general	10_15141188	10	15,141,188	A	G	3	-2.79	18.09
Early	general	10_15141188	10	15,141,188	A	G	10	-1.30	18.09
Early	general	10_23840886	10	23,840,886	A	G	2	-6.01	22.09
Early	general	10_23840886	10	23,840,886	A	G	3	-0.78	22.09
Early	general	10_23840886	10	23,840,886	A	G	4	-0.32	22.09
Early	general	10_23840886	10	23,840,886	A	G	5	-0.07	22.09
Early	general	10_23840886	10	23,840,886	A	G	6	-0.52	22.09
Early	general	10_23840886	10	23,840,886	A	G	7	0.29	22.09
Early	general	10_23840886	10	23,840,886	A	G	8	-0.57	22.09
Early	general	10_23840886	10	23,840,886	A	G	9	-4.17	22.09
Early	general	10_23840886	10	23,840,886	A	G	10	-0.21	22.09
Early	general	2_29103764	2	29,103,764	C	T	0	-2.79	20.31
Early	general	2_29103764	2	29,103,764	C	T	1	-2.99	20.31
Early	general	2_29103764	2	29,103,764	C	T	2	-1.64	20.31
Early	general	2_29103764	2	29,103,764	C	T	5	-1.74	20.31
Early	general	2_29103764	2	29,103,764	C	T	8	-3.22	20.31
Early	general	2_29103764	2	29,103,764	C	T	10	-0.11	20.31
Early	general	2_50140782	2	50,140,782	A	G	7	44.50	16.78
Early	general	2_50140782	2	50,140,782	A	G	8	1.22	16.78
Early	general	2_50140782	2	50,140,782	A	G	9	4.14	16.78
Early	general	2_50140782	2	50,140,782	A	G	10	3.25	16.78
Early	general	2_52421397	2	52,421,397	C	T	7	-8.80	13.43
Early	general	2_52421397	2	52,421,397	C	T	8	0.54	13.43
Early	general	2_52421397	2	52,421,397	C	T	9	-2.08	13.43
Early	general	2_52421397	2	52,421,397	C	T	10	-0.50	13.43
Early	general	4_10932960	4	10,932,960	A	C	6	7.34	15.30
Early	general	4_10932960	4	10,932,960	A	C	7	-0.83	15.30
Early	general	4_10932960	4	10,932,960	A	C	8	-2.02	15.30
Early	general	4_10932960	4	10,932,960	A	C	9	-1.28	15.30
Early	general	4_10932960	4	10,932,960	A	C	10	0.70	15.30
Early	general	5_24180796	5	24,180,796	A	G	0	-2.82	18.82
Early	general	5_24180796	5	24,180,796	A	G	1	-3.04	18.82
Early	general	5_24180796	5	24,180,796	A	G	2	-1.47	18.82
Early	general	5_24180796	5	24,180,796	A	G	3	-3.00	18.82
Early	general	5_24180796	5	24,180,796	A	G	4	-2.95	18.82
Early	general	5_24180796	5	24,180,796	A	G	6	-2.05	18.82
Early	general	5_24180796	5	24,180,796	A	G	10	0.07	18.82
Early	general	5_27588687	5	27,588,687	G	T	0	-2.61	18.71
Early	general	5_27588687	5	27,588,687	G	T	1	-1.37	18.71
Early	general	5_27588687	5	27,588,687	G	T	2	-4.84	18.71
Early	general	5_27588687	5	27,588,687	G	T	3	0.77	18.71
Early	general	5_27588687	5	27,588,687	G	T	4	0.47	18.71
Early	general	5_27588687	5	27,588,687	G	T	10	-0.44	18.71
Early	general	5_9031549	5	9,031,549	G	T	7	35.17	13.80
Early	general	5_9031549	5	9,031,549	G	T	8	1.47	13.80
Early	general	5_9031549	5	9,031,549	G	T	9	2.87	13.80
Early	general	5_9031549	5	9,031,549	G	T	10	2.64	13.80
Early	general	6_19116741	6	19,116,741	A	T	2	-2.94	19.17
Early	general	6_19116741	6	19,116,741	A	T	3	-0.04	19.17
Early	general	6_19116741	6	19,116,741	A	T	4	-0.20	19.17
Early	general	6_19116741	6	19,116,741	A	T	5	-0.14	19.17
Early	general	6_19116741	6	19,116,741	A	T	6	-0.08	19.17
Early	general	6_19116741	6	19,116,741	A	T	7	0.43	19.17
Early	general	6_19116741	6	19,116,741	A	T	8	-0.84	19.17
Early	general	6_19116741	6	19,116,741	A	T	9	-3.95	19.17
Early	general	6_19116741	6	19,116,741	A	T	10	0.12	19.17
Early	general	9_33845426	9	33,845,426	C	T	0	-1.51	20.33
Early	general	9_33845426	9	33,845,426	C	T	1	1.25	20.33
Early	general	9_33845426	9	33,845,426	C	T	2	-4.83	20.33
Early	general	9_33845426	9	33,845,426	C	T	3	-2.30	20.33
Early	general	9_33845426	9	33,845,426	C	T	4	-1.68	20.33
Early	general	9_33845426	9	33,845,426	C	T	6	-2.02	20.33
Early	general	9_33845426	9	33,845,426	C	T	10	0.17	20.33
Early	general	contig_28124_7059	contig_28124	7059	C	G	0	-1.11	21.59
Early	general	contig_28124_7059	contig_28124	7059	C	G	1	1.28	21.59
Early	general	contig_28124_7059	contig_28124	7059	C	G	2	-4.76	21.59
Early	general	contig_28124_7059	contig_28124	7059	C	G	3	-2.04	21.59
Early	general	contig_28124_7059	contig_28124	7059	C	G	5	-2.18	21.59
Early	general	contig_28124_7059	contig_28124	7059	C	G	6	-2.08	21.59
Early	general	contig_28124_7059	contig_28124	7059	C	G	8	-2.05	21.59
Early	general	contig_28124_7059	contig_28124	7059	C	G	10	0.36	21.59
Early	general	contig_32272_9677	contig_32272	9677	A	G	7	43.04	14.59
Early	general	contig_32272_9677	contig_32272	9677	A	G	8	2.78	14.59
Early	general	contig_32272_9677	contig_32272	9677	A	G	9	3.37	14.59
Early	general	contig_32272_9677	contig_32272	9677	A	G	10	3.17	14.59
Early	general	contig_36570_19390	contig_36570	19,390	C	T	0	-1.29	18.56
Early	general	contig_36570_19390	contig_36570	19,390	C	T	1	1.28	18.56
Early	general	contig_36570_19390	contig_36570	19,390	C	T	2	1.08	18.56
Early	general	contig_36570_19390	contig_36570	19,390	C	T	3	2.12	18.56
Early	general	contig_36570_19390	contig_36570	19,390	C	T	4	-3.77	18.56
Early	general	contig_36570_19390	contig_36570	19,390	C	T	6	-1.16	18.56
Early	general	contig_36570_19390	contig_36570	19,390	C	T	10	0.06	18.56
Early	general	contig_36570_19433	contig_36570	19,433	C	T	0	-1.90	20.03
Early	general	contig_36570_19433	contig_36570	19,433	C	T	1	-0.68	20.03
Early	general	contig_36570_19433	contig_36570	19,433	C	T	2	-1.29	20.03
Early	general	contig_36570_19433	contig_36570	19,433	C	T	3	-2.15	20.03
Early	general	contig_36570_19433	contig_36570	19,433	C	T	4	-3.30	20.03
Early	general	contig_36570_19433	contig_36570	19,433	C	T	6	0.99	20.03
Early	general	contig_36570_19433	contig_36570	19,433	C	T	10	0.19	20.03
Early	general	contig_36746_108753	contig_36746	108,753	G	T	0	-1.34	24.61
Early	general	contig_36746_108753	contig_36746	108,753	G	T	1	-1.91	24.61
Early	general	contig_36746_108753	contig_36746	108,753	G	T	2	-4.65	24.61
Early	general	contig_36746_108753	contig_36746	108,753	G	T	3	2.09	24.61
Early	general	contig_36746_108753	contig_36746	108,753	G	T	4	2.38	24.61
Early	general	contig_36746_108753	contig_36746	108,753	G	T	5	-1.27	24.61
Early	general	contig_36746_108753	contig_36746	108,753	G	T	7	-1.17	24.61
Early	general	contig_36746_108753	contig_36746	108,753	G	T	10	-0.03	24.61
Early	general	contig_36908_12883	contig_36908	12,883	C	T	0	-1.26	23.61
Early	general	contig_36908_12883	contig_36908	12,883	C	T	1	1.73	23.61
Early	general	contig_36908_12883	contig_36908	12,883	C	T	2	-2.03	23.61
Early	general	contig_36908_12883	contig_36908	12,883	C	T	3	-1.85	23.61
Early	general	contig_36908_12883	contig_36908	12,883	C	T	10	0.07	23.61
Early	general	contig_39315_31775	contig_39315	31,775	A	G	4	27.22	17.50
Early	general	contig_39315_31775	contig_39315	31,775	A	G	5	-0.03	17.50
Early	general	contig_39315_31775	contig_39315	31,775	A	G	6	0.24	17.50
Early	general	contig_39315_31775	contig_39315	31,775	A	G	7	1.68	17.50
Early	general	contig_39315_31775	contig_39315	31,775	A	G	8	1.88	17.50
Early	general	contig_39315_31775	contig_39315	31,775	A	G	9	2.33	17.50
Early	general	contig_39315_31775	contig_39315	31,775	A	G	10	2.18	17.50
Early	general	contig_50499_5072	contig_50499	5072	C	T	5	30.43	20.20
Early	general	contig_50499_5072	contig_50499	5072	C	T	6	0.31	20.20
Early	general	contig_50499_5072	contig_50499	5072	C	T	7	-0.09	20.20
Early	general	contig_50499_5072	contig_50499	5072	C	T	8	1.08	20.20
Early	general	contig_50499_5072	contig_50499	5072	C	T	9	-0.53	20.20
Early	general	contig_50499_5072	contig_50499	5072	C	T	10	2.34	20.20
Early	general	contig_65540_9617	contig_65540	9617	C	T	7	18.41	14.46
Early	general	contig_65540_9617	contig_65540	9617	C	T	8	0.59	14.46
Early	general	contig_65540_9617	contig_65540	9617	C	T	9	1.67	14.46
Early	general	contig_65540_9617	contig_65540	9617	C	T	10	1.51	14.46
Normal	1-dom-alt	1_17142404	1	17,142,404	A	T		-1.43	9.95
Normal	1-dom-alt	4_55767476	4	55,767,476	C	T		-0.99	9.80
Normal	1-dom-alt	6_22694411	6	22,694,411	G	T		1.27	11.66
Normal	1-dom-alt	6_22694443	6	22,694,443	C	G		1.18	10.55
Normal	1-dom-ref	1_12085189	1	12,085,189	A	G		1.13	15.42
Normal	1-dom-ref	1_12538127	1	12,538,127	C	T		1.36	11.86
Normal	1-dom-ref	1_63454860	1	63,454,860	C	T		1.28	14.21
Normal	1-dom-ref	10_33837431	10	33,837,431	A	G		1.78	15.19
Normal	1-dom-ref	4_15026970	4	15,026,970	C	T		0.81	13.97
Normal	1-dom-ref	5_2283521	5	2,283,521	A	G		-1.57	14.18
Normal	1-dom-ref	5_7139189	5	7,139,189	A	C		1.34	13.12
Normal	1-dom-ref	contig_18195_75278	contig_18195	75,278	G	T		1.41	14.14
Normal	2-dom-alt	6_38041421	6	38,041,421	C	T		1.48	15.00
Normal	2-dom-ref	2_28319736	2	28,319,736	C	T		-1.57	18.83
Normal	2-dom-ref	6_32116508	6	32,116,508	G	T		1.40	13.04
Normal	2-dom-ref	7_4304903	7	4,304,903	C	T		1.45	15.64
Normal	2-dom-ref	contig_31807_21402	contig_31807	21,402	G	T		-0.76	16.59
Normal	2-dom-ref	contig_40813_18644	contig_40813	18,644	A	G		-1.15	14.48
Normal	2-dom-ref	contig_44354_6395	contig_44354	6395	G	T		1.33	10.30
Normal	3-dom-ref	1_57631778	1	57,631,778	C	T		1.62	10.60
Normal	3-dom-ref	2_36645411	2	36,645,411	A	G		-0.95	13.47
Normal	3-dom-ref	3_9262308	3	9,262,308	A	T		0.82	14.72
Normal	3-dom-ref	7_11722177	7	11,722,177	A	T		-1.62	11.01
Normal	4-dom-alt	3_57539943	3	57,539,943	A	G		1.52	17.34
Normal	4-dom-ref	10_26811308	10	26,811,308	G	T		1.23	11.33
Normal	4-dom-ref	contig_39315_31775	contig_39315	31,775	A	G		1.68	13.14
Normal	5-dom-ref	6_32163815	6	32,163,815	A	C		1.43	12.19
Normal	general	1_112769	1	112,769	C	G	7	40.45	19.47
Normal	general	1_112769	1	112,769	C	G	8	0.65	19.47
Normal	general	1_112769	1	112,769	C	G	9	2.61	19.47
Normal	general	1_112769	1	112,769	C	G	10	3.03	19.47
Normal	general	1_38958511	1	38,958,511	A	G	7	27.13	16.20
Normal	general	1_38958511	1	38,958,511	A	G	8	-0.48	16.20
Normal	general	1_38958511	1	38,958,511	A	G	9	2.23	16.20
Normal	general	1_38958511	1	38,958,511	A	G	10	2.02	16.20
Normal	general	1_44113121	1	44,113,121	A	G	0	-1.51	21.26
Normal	general	1_44113121	1	44,113,121	A	G	1	0.71	21.26
Normal	general	1_44113121	1	44,113,121	A	G	2	-0.97	21.26
Normal	general	1_44113121	1	44,113,121	A	G	3	-2.58	21.26
Normal	general	1_44113121	1	44,113,121	A	G	4	-1.60	21.26
Normal	general	1_44113121	1	44,113,121	A	G	5	1.59	21.26
Normal	general	1_44113121	1	44,113,121	A	G	10	-0.04	21.26
Normal	general	1_61256982	1	61,256,982	C	T	0	-2.28	20.95
Normal	general	1_61256982	1	61,256,982	C	T	1	-2.70	20.95
Normal	general	1_61256982	1	61,256,982	C	T	2	-1.02	20.95
Normal	general	1_61256982	1	61,256,982	C	T	3	-2.66	20.95
Normal	general	1_61256982	1	61,256,982	C	T	10	-0.07	20.95
Normal	general	1_83853683	1	83,853,683	C	T	0	-1.34	19.33
Normal	general	1_83853683	1	83,853,683	C	T	1	0.02	19.33
Normal	general	1_83853683	1	83,853,683	C	T	2	-2.97	19.33
Normal	general	1_83853683	1	83,853,683	C	T	3	-2.10	19.33
Normal	general	1_83853683	1	83,853,683	C	T	4	-2.23	19.33
Normal	general	1_83853683	1	83,853,683	C	T	10	0.12	19.33
Normal	general	1_86961880	1	86,961,880	A	G	0	-2.57	21.34
Normal	general	1_86961880	1	86,961,880	A	G	1	-1.91	21.34
Normal	general	1_86961880	1	86,961,880	A	G	2	-1.76	21.34
Normal	general	1_86961880	1	86,961,880	A	G	3	-1.87	21.34
Normal	general	1_86961880	1	86,961,880	A	G	10	0.09	21.34
Normal	general	2_29103764	2	29,103,764	C	T	0	-1.49	18.16
Normal	general	2_29103764	2	29,103,764	C	T	1	-2.27	18.16
Normal	general	2_29103764	2	29,103,764	C	T	2	-2.35	18.16
Normal	general	2_29103764	2	29,103,764	C	T	5	-1.43	18.16
Normal	general	2_29103764	2	29,103,764	C	T	8	-2.64	18.16
Normal	general	2_29103764	2	29,103,764	C	T	10	0.14	18.16
Normal	general	2_47076601	2	47,076,601	A	G	0	-2.68	17.11
Normal	general	2_47076601	2	47,076,601	A	G	1	-0.11	17.11
Normal	general	2_47076601	2	47,076,601	A	G	2	-2.06	17.11
Normal	general	2_47076601	2	47,076,601	A	G	10	-0.44	17.11
Normal	general	2_50120048	2	50,120,048	G	T	6	2.44	17.64
Normal	general	2_50120048	2	50,120,048	G	T	7	-1.05	17.64
Normal	general	2_50120048	2	50,120,048	G	T	8	0.68	17.64
Normal	general	2_50120048	2	50,120,048	G	T	9	-0.71	17.64
Normal	general	2_50120048	2	50,120,048	G	T	10	0.32	17.64
Normal	general	2_9007388	2	9,007,388	G	T	5	9.06	20.77
Normal	general	2_9007388	2	9,007,388	G	T	6	-0.04	20.77
Normal	general	2_9007388	2	9,007,388	G	T	7	0.59	20.77
Normal	general	2_9007388	2	9,007,388	G	T	8	1.22	20.77
Normal	general	2_9007388	2	9,007,388	G	T	9	0.65	20.77
Normal	general	2_9007388	2	9,007,388	G	T	10	0.52	20.77
Normal	general	3_1957031	3	1,957,031	A	G	7	29.33	14.63
Normal	general	3_1957031	3	1,957,031	A	G	8	0.37	14.63
Normal	general	3_1957031	3	1,957,031	A	G	9	0.63	14.63
Normal	general	3_1957031	3	1,957,031	A	G	10	2.24	14.63
Normal	general	3_43581242	3	43,581,242	A	G	0	7.67	23.25
Normal	general	3_43581242	3	43,581,242	A	G	1	0.68	23.25
Normal	general	3_43581242	3	43,581,242	A	G	3	-0.03	23.25
Normal	general	3_43581242	3	43,581,242	A	G	4	-0.34	23.25
Normal	general	3_43581242	3	43,581,242	A	G	5	0.01	23.25
Normal	general	3_43581242	3	43,581,242	A	G	6	-0.02	23.25
Normal	general	3_43581242	3	43,581,242	A	G	7	1.24	23.25
Normal	general	3_43581242	3	43,581,242	A	G	8	0.20	23.25
Normal	general	3_43581242	3	43,581,242	A	G	9	-2.31	23.25
Normal	general	3_43581242	3	43,581,242	A	G	10	0.75	23.25
Normal	general	3_57938489	3	57,938,489	A	G	5	8.20	18.13
Normal	general	3_57938489	3	57,938,489	A	G	6	-2.31	18.13
Normal	general	3_57938489	3	57,938,489	A	G	7	-0.02	18.13
Normal	general	3_57938489	3	57,938,489	A	G	8	0.91	18.13
Normal	general	3_57938489	3	57,938,489	A	G	9	0.74	18.13
Normal	general	3_57938489	3	57,938,489	A	G	10	0.57	18.13
Normal	general	4_10932960	4	10,932,960	A	C	6	-18.11	14.98
Normal	general	4_10932960	4	10,932,960	A	C	7	-1.46	14.98
Normal	general	4_10932960	4	10,932,960	A	C	8	-3.31	14.98
Normal	general	4_10932960	4	10,932,960	A	C	9	-2.73	14.98
Normal	general	4_10932960	4	10,932,960	A	C	10	-1.16	14.98
Normal	general	4_28389065	4	28,389,065	A	G	0	-2.58	18.29
Normal	general	4_28389065	4	28,389,065	A	G	1	-1.07	18.29
Normal	general	4_28389065	4	28,389,065	A	G	2	0.26	18.29
Normal	general	4_28389065	4	28,389,065	A	G	3	-2.88	18.29
Normal	general	4_28389065	4	28,389,065	A	G	10	-0.73	18.29
Normal	general	4_49815442	4	49,815,442	A	C	0	-2.73	17.12
Normal	general	4_49815442	4	49,815,442	A	C	1	-1.18	17.12
Normal	general	4_49815442	4	49,815,442	A	C	2	-1.30	17.12
Normal	general	4_49815442	4	49,815,442	A	C	10	0.04	17.12
Normal	general	4_55115204	4	55,115,204	C	G	5	8.69	17.70
Normal	general	4_55115204	4	55,115,204	C	G	7	-0.41	17.70
Normal	general	4_55115204	4	55,115,204	C	G	8	-0.42	17.70
Normal	general	4_55115204	4	55,115,204	C	G	9	-0.99	17.70
Normal	general	4_55115204	4	55,115,204	C	G	10	0.79	17.70
Normal	general	5_20661488	5	20,661,488	A	G	8	42.50	15.03
Normal	general	5_20661488	5	20,661,488	A	G	9	2.83	15.03
Normal	general	5_20661488	5	20,661,488	A	G	10	3.13	15.03
Normal	general	8_13879675	8	13,879,675	C	T	0	-1.62	21.32
Normal	general	8_13879675	8	13,879,675	C	T	1	-0.85	21.32
Normal	general	8_13879675	8	13,879,675	C	T	2	-2.41	21.32
Normal	general	8_13879675	8	13,879,675	C	T	3	-1.21	21.32
Normal	general	8_13879675	8	13,879,675	C	T	10	0.73	21.32
Normal	general	8_13879677	8	13,879,677	A	G	7	17.10	18.62
Normal	general	8_13879677	8	13,879,677	A	G	8	-0.67	18.62
Normal	general	8_13879677	8	13,879,677	A	G	9	0.05	18.62
Normal	general	8_13879677	8	13,879,677	A	G	10	1.38	18.62
Normal	general	8_21985103	8	21,985,103	A	C	0	-0.83	18.15
Normal	general	8_21985103	8	21,985,103	A	C	1	-0.99	18.15
Normal	general	8_21985103	8	21,985,103	A	C	2	-0.53	18.15
Normal	general	8_21985103	8	21,985,103	A	C	10	0.78	18.15
Normal	general	8_23373494	8	23,373,494	A	C	7	31.29	16.13
Normal	general	8_23373494	8	23,373,494	A	C	8	0.61	16.13
Normal	general	8_23373494	8	23,373,494	A	C	9	2.79	16.13
Normal	general	8_23373494	8	23,373,494	A	C	10	2.32	16.13
Normal	general	9_29313099	9	29,313,099	C	T	2	31.62	21.84
Normal	general	9_29313099	9	29,313,099	C	T	3	1.83	21.84
Normal	general	9_29313099	9	29,313,099	C	T	4	2.52	21.84
Normal	general	9_29313099	9	29,313,099	C	T	5	2.29	21.84
Normal	general	9_29313099	9	29,313,099	C	T	6	2.27	21.84
Normal	general	9_29313099	9	29,313,099	C	T	7	2.46	21.84
Normal	general	9_29313099	9	29,313,099	C	T	8	2.58	21.84
Normal	general	9_29313099	9	29,313,099	C	T	9	2.03	21.84
Normal	general	9_29313099	9	29,313,099	C	T	10	2.33	21.84
Normal	general	9_33845426	9	33,845,426	C	T	0	-1.22	20.65
Normal	general	9_33845426	9	33,845,426	C	T	1	1.26	20.65
Normal	general	9_33845426	9	33,845,426	C	T	2	-3.55	20.65
Normal	general	9_33845426	9	33,845,426	C	T	3	-2.44	20.65
Normal	general	9_33845426	9	33,845,426	C	T	4	-1.97	20.65
Normal	general	9_33845426	9	33,845,426	C	T	6	-2.86	20.65
Normal	general	9_33845426	9	33,845,426	C	T	10	0.14	20.65
Normal	general	contig_28124_7059	contig_28124	7059	C	G	0	-0.67	21.72
Normal	general	contig_28124_7059	contig_28124	7059	C	G	1	1.34	21.72
Normal	general	contig_28124_7059	contig_28124	7059	C	G	2	-3.41	21.72
Normal	general	contig_28124_7059	contig_28124	7059	C	G	3	-2.86	21.72
Normal	general	contig_28124_7059	contig_28124	7059	C	G	5	-2.15	21.72
Normal	general	contig_28124_7059	contig_28124	7059	C	G	6	-1.54	21.72
Normal	general	contig_28124_7059	contig_28124	7059	C	G	8	-1.62	21.72
Normal	general	contig_28124_7059	contig_28124	7059	C	G	10	0.34	21.72
Normal	general	contig_35710_3625	contig_35710	3625	G	T	7	21.73	13.46
Normal	general	contig_35710_3625	contig_35710	3625	G	T	8	-0.09	13.46
Normal	general	contig_35710_3625	contig_35710	3625	G	T	9	2.48	13.46
Normal	general	contig_35710_3625	contig_35710	3625	G	T	10	1.66	13.46
Normal	general	contig_36359_81087	contig_36359	81,087	A	G	0	-1.85	22.31
Normal	general	contig_36359_81087	contig_36359	81,087	A	G	1	-0.60	22.31
Normal	general	contig_36359_81087	contig_36359	81,087	A	G	2	-3.01	22.31
Normal	general	contig_36359_81087	contig_36359	81,087	A	G	3	-2.45	22.31
Normal	general	contig_36359_81087	contig_36359	81,087	A	G	4	0.50	22.31
Normal	general	contig_36359_81087	contig_36359	81,087	A	G	5	0.98	22.31
Normal	general	contig_36359_81087	contig_36359	81,087	A	G	6	-1.22	22.31
Normal	general	contig_36359_81087	contig_36359	81,087	A	G	9	-1.72	22.31
Normal	general	contig_36359_81087	contig_36359	81,087	A	G	10	-0.02	22.31
Normal	general	contig_36570_19433	contig_36570	19,433	C	T	0	-2.60	20.51
Normal	general	contig_36570_19433	contig_36570	19,433	C	T	1	-1.32	20.51
Normal	general	contig_36570_19433	contig_36570	19,433	C	T	2	-1.27	20.51
Normal	general	contig_36570_19433	contig_36570	19,433	C	T	3	-1.63	20.51
Normal	general	contig_36570_19433	contig_36570	19,433	C	T	4	-2.95	20.51
Normal	general	contig_36570_19433	contig_36570	19,433	C	T	6	0.64	20.51
Normal	general	contig_36570_19433	contig_36570	19,433	C	T	10	-0.21	20.51
Normal	general	contig_36908_12883	contig_36908	12,883	C	T	0	-1.32	25.39
Normal	general	contig_36908_12883	contig_36908	12,883	C	T	1	1.10	25.39
Normal	general	contig_36908_12883	contig_36908	12,883	C	T	2	-2.02	25.39
Normal	general	contig_36908_12883	contig_36908	12,883	C	T	3	-2.48	25.39
Normal	general	contig_36908_12883	contig_36908	12,883	C	T	10	-0.06	25.39
Normal	general	contig_38146_20675	contig_38146	20,675	G	T	0	-1.39	19.31
Normal	general	contig_38146_20675	contig_38146	20,675	G	T	1	0.21	19.31
Normal	general	contig_38146_20675	contig_38146	20,675	G	T	2	-0.29	19.31
Normal	general	contig_38146_20675	contig_38146	20,675	G	T	3	0.24	19.31
Normal	general	contig_38146_20675	contig_38146	20,675	G	T	4	-2.08	19.31
Normal	general	contig_38146_20675	contig_38146	20,675	G	T	10	0.10	19.31
Normal	general	contig_39799_65	contig_39799	65	C	T	7	22.12	17.07
Normal	general	contig_39799_65	contig_39799	65	C	T	8	0.18	17.07
Normal	general	contig_39799_65	contig_39799	65	C	T	9	2.78	17.07
Normal	general	contig_39799_65	contig_39799	65	C	T	10	1.69	17.07
Normal	general	contig_44982_8900	contig_44982	8900	C	T	0	-2.19	21.47
Normal	general	contig_44982_8900	contig_44982	8900	C	T	1	0.74	21.47
Normal	general	contig_44982_8900	contig_44982	8900	C	T	2	-2.12	21.47
Normal	general	contig_44982_8900	contig_44982	8900	C	T	3	-2.58	21.47
Normal	general	contig_44982_8900	contig_44982	8900	C	T	4	-2.27	21.47
Normal	general	contig_44982_8900	contig_44982	8900	C	T	10	-0.18	21.47
Normal	general	contig_50499_5072	contig_50499	5072	C	T	5	25.17	19.34
Normal	general	contig_50499_5072	contig_50499	5072	C	T	6	0.10	19.34
Normal	general	contig_50499_5072	contig_50499	5072	C	T	7	0.12	19.34
Normal	general	contig_50499_5072	contig_50499	5072	C	T	8	0.50	19.34
Normal	general	contig_50499_5072	contig_50499	5072	C	T	9	-0.26	19.34
Normal	general	contig_50499_5072	contig_50499	5072	C	T	10	1.93	19.34
Normal	general	contig_59437_13870	contig_59437	13,870	C	T	2	38.80	22.14
Normal	general	contig_59437_13870	contig_59437	13,870	C	T	4	0.90	22.14
Normal	general	contig_59437_13870	contig_59437	13,870	C	T	5	1.09	22.14
Normal	general	contig_59437_13870	contig_59437	13,870	C	T	6	2.87	22.14
Normal	general	contig_59437_13870	contig_59437	13,870	C	T	7	2.82	22.14
Normal	general	contig_59437_13870	contig_59437	13,870	C	T	8	3.04	22.14
Normal	general	contig_59437_13870	contig_59437	13,870	C	T	9	3.11	22.14
Normal	general	contig_59437_13870	contig_59437	13,870	C	T	10	2.67	22.14

### Candidate genes

A total of 4757 genes were found within the LD region of the 77 markers, with 1695 having a known function in plants. From the 1695 candidate genes, 82 were associated with sucrose accumulation and/or sucrose production and were within the LD region of 39 of the 77 associated markers (Table 5). For the remaining 38 markers, there were no candidate genes with an annotated function related to sucrose accumulation and/or production, and therefore they were not considered for further analysis. From the 82 candidate genes, eight were involved in sucrose transport, seven were involved in starch and sucrose metabolism in *Sorghum* sp [37–39]. , and 67 were glycosyltransferases, glucosidases, hormones, and transcription factors (Table 5).

**Table 5:** Candidate genes in the LD region window (i.e., 500 Kb upstream and downstream) from 39 of the 77 markers associated with the accumulation and/or production of sucrose at early (10 map), normal (13 map), or shared between both maturities

Maturity	Marker	Chromosome	Position (bp)	Reference allele	Alternative allele	Candidate gene
Both	1_61256982	1	61,256,982	C	T	Similar to At5g38460: Probable dolichyl pyrophosphate Man9GlcNAc2 alpha-1%2C3-glucosyltransferase (Arabidopsis thaliana)
Both	1_61256982	1	61,256,982	C	T	Similar to GLU1: Endoglucanase 9 (Oryza sativa subsp. japonica)
Both	1_61256982	1	61,256,982	C	T	Similar to MST1: Sugar transport protein MST1 (Oryza sativa subsp. japonica)
Both	1_86961880	1	86,961,880	A	G	Similar to FKGP: Bifunctional fucokinase/fucose pyrophosphorylase (Arabidopsis thaliana)
Both	2_29103764	2	29,103,764	C	T	Similar to At1g05030: Probable plastidic glucose transporter 1 (Arabidopsis thaliana)
Both	2_29103764	2	29,103,764	C	T	Similar to TPS6: Alpha%2Calpha-trehalose-phosphate synthase [UDP-forming] 6 (Arabidopsis thaliana)
Both	3_57539943	3	57,539,943	A	G	Similar to ERF112: Ethylene-responsive transcription factor ERF112 (Arabidopsis thaliana)
Both	3_57539943	3	57,539,943	A	G	Similar to FAB1B: 1-phosphatidylinositol-3-phosphate 5-kinase FAB1B (Arabidopsis thaliana)
Both	3_57539943	3	57,539,943	A	G	Similar to PSI1: Protein PSK SIMULATOR 1 (Arabidopsis thaliana)
Both	4_55115204	4	55,115,204	C	G	Similar to SUT4: Sucrose transport protein SUT4 (Oryza sativa subsp. japonica)
Both	9_33845426	9	33,845,426	C	T	Similar to ERD6-like 4: Sugar transporter ERD6-like 4
Both	9_33845426	9	33,845,426	C	T	Similar to Glucose-1-phosphate adenylyltransferase large subunit 3%2 C chloroplastic/amyloplastic
Early	1_33380771	1	33,380,771	A	C	Similar to ABCG50: ABC transporter G family member 50 (Oryza sativa subsp. japonica)
Early	1_33380771	1	33,380,771	A	C	Similar to MST2: Sugar transport protein MST2 (Oryza sativa subsp. japonica)
Early	10_1273014	10	1,273,014	A	C	Similar to At2g16790: Gluconokinase (Arabidopsis thaliana)
Early	10_1273014	10	1,273,014	A	C	Similar to CGT: UDP-glucose:2-hydroxyflavanone C-glucosyltransferase (Oryza sativa subsp. japonica)
Early	10_1273014	10	1,273,014	A	C	Similar to FLZ1: FCS-Like Zinc finger 1 (Arabidopsis thaliana)
Early	10_1273014	10	1,273,014	A	C	Similar to G6PGH1: 6-phosphogluconate dehydrogenase%2 C decarboxylating 1 (Oryza sativa subsp. japonica)
Early	10_1273014	10	1,273,014	A	C	Similar to PIN1A: Auxin efflux carrier component 1a (Oryza sativa subsp. japonica)
Early	10_1273014	10	1,273,014	A	C	Similar to Putative invertase inhibitor (Platanus acerifolia)
Early	10_1273014	10	1,273,014	A	C	Similar to SIS8: Probable serine/threonine-protein kinase SIS8 (Arabidopsis thaliana)
Early	10_1273014	10	1,273,014	A	C	Similar to UGT72B3: UDP-glycosyltransferase 72B3 (Arabidopsis thaliana)
Early	10_15141188	10	15,141,188	A	G	Similar to GLU13: Endoglucanase 17 (Oryza sativa subsp. japonica)
Early	10_23840886	10	23,840,886	A	G	Similar to CESA11: Putative cellulose synthase A catalytic subunit 11 [UDP-forming] (Oryza sativa subsp. japonica)
Early	10_23840886	10	23,840,886	A	G	Similar to IAA23: Auxin-responsive protein IAA23 (Oryza sativa subsp. japonica)
Early	10_23840886	10	23,840,886	A	G	Similar to WIN1: Ethylene-responsive transcription factor WIN1 (Arabidopsis thaliana)
Early	2_50140782	2	50,140,782	A	G	Similar to PGR: Protein PGR (Arabidopsis thaliana)
Early	2_52421397	2	52,421,397	C	T	Similar to AGPS2: Glucose-1-phosphate adenylyltransferase small subunit 2%2 C chloroplastic/amyloplastic/cytosolic (Oryza sativa subsp. japonica)
Early	2_52421397	2	52,421,397	C	T	Similar to CSTLP2: CMP-sialic acid transporter 2 (Oryza sativa subsp. japonica)
Early	2_52421397	2	52,421,397	C	T	Similar to PFP-ALPHA: Pyrophosphate--fructose 6-phosphate 1-phosphotransferase subunit alpha (Ricinus communis)
Early	3_20905930	3	20,905,930	A	T	Similar to Os01g0276800: Glucosidase 2 subunit beta (Oryza sativa subsp. japonica)
Early	3_20905930	3	20,905,930	A	T	Similar to Pyruvate kinase%2 C cytosolic isozyme (Nicotiana tabacum)
Early	5_24180796	5	24,180,796	A	G	Similar to EOL1: ETO1-like protein 1 (Arabidopsis thaliana)
Early	5_24180796	5	24,180,796	A	G	Similar to LTPG19: Non-specific lipid transfer protein GPI-anchored 19 (Arabidopsis thaliana)
Early	5_27588687	5	27,588,687	G	T	Similar to CPS1: Ent-copalyl diphosphate synthase 1%2 C chloroplastic (Oryza sativa subsp. japonica)
Early	5_9031549	5	9,031,549	G	T	Similar to Alkaline/neutral invertase CINV2
Early	5_9031549	5	9,031,549	G	T	Similar to Cytosolic invertase 1
Early	6_19116741	6	19,116,741	A	T	Similar to ALG10: Dol-P-Glc: Glc(2)Man(9)GlcNAc(2)-PP-Dol alpha-1%2C2-glucosyltransferase (Arabidopsis thaliana)
Early	6_19116741	6	19,116,741	A	T	Similar to ATG10: Ubiquitin-like-conjugating enzyme ATG10 (Arabidopsis thaliana)
Early	6_19116741	6	19,116,741	A	T	Similar to TBL2: Protein trichome birefringence-like 2 (Arabidopsis thaliana)
Normal	1_12085189	1	12,085,189	A	G	Similar to GLO1: Peroxisomal (S)-2-hydroxy-acid oxidase GLO1 (Oryza sativa subsp. japonica)
Normal	1_12085189	1	12,085,189	A	G	Similar to PIL13: Transcription factor PHYTOCHROME INTERACTING FACTOR-LIKE 13 (Oryza sativa subsp. japonica)
Normal	1_12085189	1	12,085,189	A	G	Similar to SAUR40: Auxin-responsive protein SAUR40 (Arabidopsis thaliana)
Normal	1_12085189	1	12,085,189	A	G	Similar to SYP121: Syntaxin-121 (Arabidopsis thaliana)
Normal	1_12538127	1	12,538,127	C	T	Similar to Glucose-6-phosphate isomerase%2 C cytosolic
Normal	1_17142404	1	17,142,404	A	T	Similar to At2g16220: Putative F-box protein At2g16220 (Arabidopsis thaliana)
Normal	1_17142404	1	17,142,404	A	T	Similar to At5g25310: Probable glycosyltransferase At5g25310 (Arabidopsis thaliana)
Normal	1_17142404	1	17,142,404	A	T	Similar to CIN3: Beta-fructofuranosidase%2 C insoluble isoenzyme 3 (Oryza sativa subsp. japonica)
Normal	1_17142404	1	17,142,404	A	T	Similar to DRIP2: E3 ubiquitin protein ligase DRIP2 (Arabidopsis thaliana)
Normal	1_17142404	1	17,142,404	A	T	Similar to GEM: GLABRA2 expression modulator (Arabidopsis thaliana)
Normal	1_17142404	1	17,142,404	A	T	Similar to Protein WRKY1 (Zea mays)
Normal	1_38958511	1	38,958,511	A	G	Similar to CESA7: Cellulose synthase A catalytic subunit 7 [UDP-forming] (Oryza sativa subsp. japonica)
Normal	1_38958511	1	38,958,511	A	G	Similar to KAT1: 3-ketoacyl CoA thiolase 1%2 C peroxisomal (Petunia hybrida)
Normal	1_38958511	1	38,958,511	A	G	Similar to NPF5.2: Protein NRT1/ PTR FAMILY 5.2 (Arabidopsis thaliana)
Normal	1_57631778	1	57,631,778	C	T	Similar to MYB61: Transcription factor MYB61 (Arabidopsis thaliana)
Normal	1_57631778	1	57,631,778	C	T	Similar to PDK: [Pyruvate dehydrogenase (acetyl-transferring)] kinase%2 C mitochondrial (Arabidopsis thaliana)
Normal	1_83853683	1	83,853,683	C	T	Similar to GAPB: Glyceraldehyde-3-phosphate dehydrogenase B%2 C chloroplastic (Spinacia oleracea)
Normal	10_26811308	10	26,811,308	G	T	Similar to ERF12: Ethylene-responsive transcription factor 12 (Arabidopsis thaliana)
Normal	10_26811308	10	26,811,308	G	T	Similar to PLT5: Polyol transporter 5 (Arabidopsis thaliana)
Normal	10_33837431	10	33,837,431	A	G	Similar to BGLU25: Beta-glucosidase 25 (Oryza sativa subsp. japonica)
Normal	10_33837431	10	33,837,431	A	G	Similar to IRX14: Probable beta-1%2C4-xylosyltransferase IRX14 (Oryza sativa subsp. japonica)
Normal	10_33837431	10	33,837,431	A	G	Similar to IRX15-L: Protein IRX15-LIKE (Arabidopsis thaliana)
Normal	10_33837431	10	33,837,431	A	G	Similar to PRX74: Peroxidase 1 (Oryza sativa subsp. japonica)
Normal	2_50120048	2	50,120,048	G	T	Similar to PGR: Protein PGR (Arabidopsis thaliana)
Normal	3_1957031	3	1,957,031	A	G	Similar to EIN2: Protein ETHYLENE-INSENSITIVE 2 (Oryza sativa subsp. japonica)
Normal	3_1957031	3	1,957,031	A	G	Similar to YUCCA4: Indole-3-pyruvate monooxygenase YUCCA4 (Oryza sativa subsp. japonica)
Normal	3_43581242	3	43,581,242	A	G	Similar to BC10: Glycosyltransferase BC10 (Oryza sativa subsp. japonica)
Normal	3_43581242	3	43,581,242	A	G	Similar to BURP3: BURP domain-containing protein 3 (Oryza sativa subsp. japonica)
Normal	3_43581242	3	43,581,242	A	G	Similar to OSK1: Serine/threonine protein kinase OSK1 (Oryza sativa subsp. japonica)
Normal	3_43581242	3	43,581,242	A	G	Similar to TPS7: Probable alpha%2Calpha-trehalose-phosphate synthase [UDP-forming] 7 (Arabidopsis thaliana)
Normal	3_43581242	3	43,581,242	A	G	Similar to UGT13: UDP-glycosyltransferase 13 (Pueraria montana var. lobata)
Normal	3_43581242	3	43,581,242	A	G	Similar to YUC2: Indole-3-pyruvate monooxygenase YUCCA2 (Arabidopsis thaliana)
Normal	3_57938489	3	57,938,489	A	G	Similar to Beta-galactosidase 3
Normal	3_57938489	3	57,938,489	A	G	Similar to BC10: Glycosyltransferase BC10 (Oryza sativa subsp. japonica)
Normal	4_15026970	4	15,026,970	C	T	Similar to MSSP2: Monosaccharide-sensing protein 2 (Arabidopsis thaliana)
Normal	4_28389065	4	28,389,065	A	G	Similar to At1g53660: Probable sugar phosphate/phosphate translocator At1g53660 (Arabidopsis thaliana)
Normal	4_49815442	4	49,815,442	A	C	Similar to TPS11: Probable alpha%2Calpha-trehalose-phosphate synthase [UDP-forming] 11 (Arabidopsis thaliana)
Normal	4_55767476	4	55,767,476	C	T	Similar to SUS6: Sucrose synthase 6 (Oryza sativa subsp. japonica)
Normal	5_2283521	5	2,283,521	A	G	Similar to WRI1: Ethylene-responsive transcription factor WRI1 (Arabidopsis thaliana)
Normal	6_38041421	6	38,041,421	C	T	Similar to At5g20260: Probable glycosyltransferase At5g20260 (Arabidopsis thaliana)
Normal	6_38041421	6	38,041,421	C	T	Similar to TMK3: Receptor-like kinase TMK3 (Arabidopsis thaliana)
Normal	7_4304903	7	4,304,903	C	T	Similar to GTE9: Transcription factor GTE9 (Arabidopsis thaliana)
Normal	8_13879675	8	13,879,675	C	T	Similar to HXK3: Hexokinase-3 (Oryza sativa subsp. japonica)
Normal	8_13879677	8	13,879,677	A	G	Similar to HXK3: Hexokinase-3 (Oryza sativa subsp. japonica)
Normal	contig_40813_18644	contig_40813	18,644	A	G	Similar to At5g16450: Putative 4-hydroxy-4-methyl-2-oxoglutarate aldolase 2 (Arabidopsis thaliana)

## Discussion

### Phenotypic analysis

Sucrose (%Cane) accumulation at early maturity was found to be 1.73% lower than the accumulation at normal maturity. These results were similar to those reported in Ethiopia [40], Egypt [41], and Brazil [42], where increased age at harvest increases sucrose content. Similar results were observed in a breeding population of 100 varieties which represent four improved generations spanning a 20 year node in China [6]. The differences in sucrose content between both ages are a consequence of physiological changes in the plant during its rapid vegetative growth phase (between 4 and 9 months after planting), where the photoassimilates are mainly used for cell elongation [17, 43]. On the other hand, during the maturation phase (between 9 and 13 months after planting), there is a decrease in the concentration of reducing sugars, invertases, and sucrose synthase (SuSy) and an increase in sucrose phosphate synthase (SPS) [41, 44–46]. Invertase activity is elevated in the upper internodes during the phase of rapid vegetative growth, but it starts to decrease when the maturation phase begins [20]. This balance between the enzymes of synthesis (i.e., SPS) and hydrolysis of sucrose (i.e., SuSy, reducing sugars, and invertases) leads to an increase in the accumulation of sugars in the sink organs because of a reduction in the demand for growth in the meristematic tissues [28, 47].

The variation in sucrose accumulation (%Cane) is assumed to result from genotypic effects and the interaction between the genotype and crop cycle [48]. For normal maturity, there was a heteroskedastic residual variance, with plant cane having the highest residual (${\sigma }_{e\left(PC\right)}^{2}=2.94$) and the first ratoon having the lowest (${\sigma }_{e\left(FR\right)}^{2}=0.68$) (Table 2). Similar results were reported in South Africa and Louisiana, where residual variance was the largest source of phenotypic variation for sucrose during plant cane [49–51]. Higher residuals in plant cane could be explained by poor, less-established root systems in comparison to the ratoons, where the plants have strong and well-established root systems, facilitating the establishment, germination, development, and growth of the plant [52].

### Population structure

The 220 genotypes were classified into four subpopulations (Fig. 2). The first and second subpopulations were composed of varieties mainly bred in Colombia. This varieties represent the 96.4% of the Colombian varieties planted in commercial fields. When analyzing subpopulation 1, the genotypes MZC 74–275, CP 57–603, and V 71 − 51 constitutes the most common parents, while for subpopulation 2, the common parents were POJ 2878, NA 56–79 and Co 775. The varieties MZC 74–275 and Co 775 are half-sibs with the variety POJ 2878, one of the common parents for subpopulation 2. Similarly, the variety POJ 2364 is within the pedigree of all common ancestors for both subpopulations, revealing the genetic resemblance between the two clusters. The genotypes POJ 2878 and POJ 2364 are widely used around the world because of their high productivity potential and adaptability to different environments [53]. The third subpopulation had the *S. spontaneum* genotype grouped with *S. officinarum, S. sinense, S. barberi*, and some interspecific genotypes. This grouping is consistent with the evolutionary relationships of the genus *Saccharum* spp., where the *S. spontaneum* has contributed with nearly 39% of the genome for the species *S. sinense* and *S. barberi*, and between 15 and 27.5% of the genome of modern cultivars [53–58]. The fourth subpopulation had only the genotypes S1, S132, and S3, all of them from the genus *Erianthus spp.* (Fig. 2). The genus *Erianthus* is one of the most closely related genera to *Saccharum spp.* and has been used mainly to increase biomass, vigor, ratooning ability, and tolerance to drought and waterlogging stresses [59]. Similarly, *Erianthus* spp. along with *Saccharum* spp., *Miscanthus* spp., *Miscanthidium* spp., *Pseudosorghum* spp., *Narenga* spp., and the trans*-Himalayan* species make up the Saccharum complex, which may be involved in the origins of cultivated sugarcane [59–61]. Finally, the 220 genotypes were divided into two main branches, one grouping the modern genotypes (light blue and magenta in (Fig. 2) and the other the wild species and interspecific crosses (Fig. 2), suggesting a narrow genetic background for the cultivars held at Cenicaña’s germplasm bank.

### Association analysis and candidate genes

Sucrose accumulation in sugarcane is a complex process that includes sucrose synthesis in the source tissues, transport of sugars from source to sink, energy generation, and sugar storage [62, 63]. In this study, a total of 82 candidate genes involved in sucrose accumulation and/or production were found within the LD region of 39 of the 77 associated markers. For the remaining 38 SNPs no candidate genes associated with sucrose production and/or accumulation were identified and for that were not considered for this analysis. From the 82 candidate genes, four key enzymes were identified in the process of sucrose synthesis: sucrose synthase 6 (SUS6), near the marker 4_55767476, insoluble beta-fructofuranosidase 2 C isoenzyme 3 (CIN3), near the marker 1_17142404, alkaline/neutral invertase (CINV2) and Cytosolic invertase 1 and (CINV1) near the marker 5_9031549 (Table 5). The markers 4_55767476 and 1_17142404 were significantly associated at normal maturity, with negative effects (Table 4), while the marker 5_9031549 was significantly associated at early maturity with positive effects (Table 4). Sucrose synthase has implications for cell metabolism, the production of metabolites, and the production of cell wall precursors (UDP-glucose) [64]. SUS6 catalyzes the reversible conversion of sucrose to UDP-glucose and fructose for various metabolic pathways [65], while the enzyme CIN3 cleaves the terminal nonreducing beta-fructofuranoside residues [66]. CINV2 can regulate sugar-mediated root development by controlling sucrose catabolism in root cells, while CINV1 participates in osmotic stress-induced inhibition of lateral root growth by controlling the concentration of hexose in the cells [64, 67]. These enzymes play a central role in the sucrose accumulation process, since sucrose is hydrolyzed by sucrose synthase or invertase in sink tissues and later used for cell growth, development, or sugar storage in the plant [21]. The negative effect for these markers suggests that the presence of these two enzymes affects sucrose accumulation by hydrolyzing this disaccharide into glucose and fructose, which are then absorbed in the sink tissues for consumption in the process of cell growth across the plasma membrane [68].

The second process of importance in sucrose accumulation is the transport of sugars from source to sink. Within this process, two plant gene families were found: sucrose transporters (SUTs) and monosaccharide transporters (MSTs) [69]. For early maturity, MST2, the plastidic glucose transporter At1g05030, and SUT4 were found within the LD region of the markers 1_33380771, 2_29103764, and 4_55115204, respectively (Table 5). At normal maturity, the candidate genes monosaccharide-sensing protein 2 MSSP2, polyol transporter 5 PLT5, and sugar-phosphate/phosphate translocator At1g53660 (GTP2) were found near the markers 4_15026970, 10_26811308 and 4_28389065, respectively (Table 5). On the other hand, the markers 1_61256982 and 9_33845426, shared in both maturations, close to the candidate genes MST and ERD6-like respectively. The genes MST1, MST2, GTP2, At1g05030, and PLT5, are all directly involved in the transport of monosaccharides, required compounds for various processes of plant growth, development [68], and osmotic adjustments (e.g., monosaccharide homeostasis) [70]. The ERD6-like gene may be strongly regulated in response to some developmental and environmental signals, including senescence, pathogen attack, Carbon/Nitrogen starvation, and diurnal changes in transient sugar storage in the vacuole [71]. SUT4 is involved in the transport of disaccharides, especially sucrose, the main photoassimilate transported from the source organ to the sink through the phloem [72, 73]. In this study, the markers 4_55115204, 4_15026970, and 10_26811308, within the LD region of the sugar transporters (Table 5), showed a positive effect (Table 4), that is, the presence of these markers appeared to contribute to an increase in sucrose content in the plant. On the other hand, the markers close to the candidate genes MST2 (1_33380771) and At1g05030 (2_29103764) had a negative effect (Table 4). This is to be expected given that at 10 months (early maturity), the plant is in transition from the rapid growth phase to the maturation phase, using the hexoses present in the plant mainly for vegetative growth and not for accumulation [74].

The candidate genes Alpha-2 C-alpha-trehalose-phosphate synthase 6 (TPS6), Alpha-2 C-alpha-trehalose-phosphate synthase 7 (TPS7), Alpha-2 C-alpha-trehalose-phosphate synthase 11 (TPS11) were found near the markers 2_29103764, 3_43581242, and 4_49815442, respectively (Table 5). These genes are involved in various phosphorylation processes, such as trehalose synthesis and glycolysis, which can regulate sucrose accumulation by acting on the expression of genes that code for carbohydrate metabolism and other metabolic enzymes [75]. For example, the candidate gene TPS, found at both maturities, is involved in the synthesis of trehalose-6-phosphate (T6P), which links growth and development to carbon status by exerting a negative feedback regulation on sucrose levels [76, 77]. However, the pyrophosphate-fructose 6-phosphate 1-phosphotransferase alpha (PFP-ALPHA), Glucose-6-phosphate isomerase 2 C cytosolic (PHI1) and hexokinase 3 (HXK3) genes, near markers 2_52421397, 1_12538127 and 8_1387967, respectively (Table 5), are involved in glycolysis, a required process for energy production [78]. PFP-ALFA, present in early maturity, participates in the breakdown of carbohydrates by catalyzing the reversible interconversion between fructose-6-phosphate and fructose-1,6-bisphosphate in a glycolysis intermediate [79]. PHI1 catalyzes the reversible isomerization of glucose-6-phosphate to fructose-6-phosphate, the second reaction step of glycolysis [80], while HXK3, a transferase found at normal maturity, is involved in glucose phosphorylation to produce glucose-6-phosphate, important for glycolysis and the pentose phosphate pathway [81]. The positive effect presented in the marker 8_13879677 near HXK3 (Table 4) suggests that the presence of this marker increases sucrose by storing glucose in the form of glucose-6-phosphate.

Transcription factors are proteins that bind to DNA to control genes in processes such as pentose-phosphate, glycolysis, and the metabolism of sugars and hormones [82]. The present study identifies two candidate genes: the ethylene-responsive transcription factor ERF12 and the ethylene-responsive transcription factor ERF112 (Table 5). Both genes were found during the maturation phase (i.e., normal maturity), suggesting that they can generate an increase in the enzymes sucrose synthase (SUS), invertase (INV), and sucrose phosphate synthase (SPS) [83]. It has been reported that an increase in the ethylene activity would be associated with the production of lignin and fiber, important compounds for source‒sink regulation and sucrose accumulation [83]. Finally, 16 markers were found associated with the accumulation of sucrose at early and normal maturity. These markers were close to genes that may be linked to the fundamental metabolic processes necessary for the production or accumulation of sucrose in any environment and at any developmental age. In particular, the genes Man9GlcNAc2 alpha-1-2C3-glucosyltransferase (At5g38460), endoglucanase 9 (GLU1), bifunctional fucokinase/fucose pyrophosphorylase (FKGP), ERF112, TPS6, MST1, probable plastidic glucose transporter 1, ERD6-like 4, and SUT4 could be considered housekeeping genes because they encode enzymes necessary for basic cellular metabolism on an ongoing basis [84]. For example, At5g38460 adds the first glucose residue to the lipid-linked oligosaccharide precursor for N-linked glycosylation, which is necessary for glycosylation and protein folding and their subsequent exit from the endoplasmic reticulum [85]. GLU1 affects internode elongation and cell wall components [86], while FKGP is involved in the metabolic reactivation of fucose by salvage paths into NDP-sugars and by converting fucose into GDP-fucose to be substrates for the biosynthesis of wall polysaccharides [87]. The SUT4, ERD6-like 4, and MST1 genes are sugar transporters and are generally considered synergistic genes because when sucrose reaches sink tissues, it is hydrolyzed into glucose and fructose (hexoses), which can be used for growth or storage through a sugar-coupled transporter (STP) [72]. The genes found in this study show that sucrose accumulation involves multiple metabolic pathways, such as trehalose and sucrose starch metabolism, and/or biological processes, such as glycolysis, as well as different sugar transporters, which act synergistically for plant development and for the accumulation of sucrose.

## Conclusions

In this study, the trait of sucrose concentration was dissected through a GWAS analysis under 12 genetic models (i.e., general, additive, and the dominant models from 1 to 5 dominant alleles) in a diverse sugarcane population of 220 genotypes. From the analysis, 16, 45, and 16 markers were found to be significantly associated with sucrose concentration at early maturity, normal maturity, and shared between both maturities, respectively. After candidate genes analysis, there were 82 genes within the LD region of only 39 markers with an annotated function involved with sucrose accumulation and/or production. For the remaining 38 markers, there was no annotated gene associated with the trait. Among the 82 candidate genes, 18 were highlighted because they were involved in sucrose hydrolysis (SUS6 and CIN3), sugar transport (i.e., MST1, MST2, PLT5, SUT4), phosphorylation processes (TPS genes), glycolysis (PFP-ALPHA and HXK3), and transcription factors (ERF12, and ERF112). These 39 markers will be helpful to further select favorable genetic resources for the sugarcane breeding process in Colombia. The highlight of this study is the genetic dissection of sucrose, a quantitative trait, in a decaploid organism and the identification of several molecular markers related to the accumulation or production of sucrose at different maturity phases. Finally, these results provide new insights into the molecular mechanisms involved in sucrose accumulation in sugarcane and contribute important genomic resources for future research on sucrose accumulation and/or production in humid environments in Colombia.

## Methods

### Experimental site

The experiment was planted in the humid environment of the Cauca River valley, Colombia, in fields of La Cabaña sugarcane mill located at 3° 10’ 58.44” N and 76° 21’ 7.599” E. The location had an agroecological zone 6H4, which is characterized by the presence of soils with high humidity (with excesses between 400 and 600 mm/year), with a predominance of clayey soils, fine textures, and poorly aerated conditions [26]. The experiment was conducted during the plant cane, first, and second ratoon. The field has a tropical climate with a total rainfall of 1669 mm, 1677.70 mm, and 1507.50 mm of accumulated precipitation for the plant cane, first, and second ratoon, respectively (Table 6). All harvests were done during the second rainy season of the Cauca River valley in the third week of September 2018 for the plant cane, October 2019 for the first ratoon, and October 2020 for the second ratoon. The average temperature ranged between 23.25 °C for the plant cane and 23.79 °C for the second ratoon, with a relative humidity above 80% for the 3 harvests (Table 6).

**Table 6:** Climate data observed during the plant cane, first, and second ratoon of the experiment planted in the humid environment of the Cauca River valley in fields of La Cabaña sugarcane mill

	Plant cane	First ratoon	Second ratoon
Age of harvest (months)	13.37	13.27	12.17
Total rainfall (mm)	1669	1677.7	1507.5
Minimum temperature (°C)	18.63	18.87	19.29
Maximum temperature (°C)	29.98	30.56	30.48
Average temperature (°C)	23.25	23.68	23.79
Relative humidity (%)	81.19	82.19	83.35

### Plant material

Cenicaña’s diverse panel of 220 genotypes from its sugarcane breeding program was selected for this study [88]. This diverse panel contained 98 genotypes that represents the genetic diversity of Cenicaña’s germplasm bank [89], 31 genotypes representatives from the wild species *Saccharum officinarum, Saccharum barberi, Saccharum sinense, Saccharum spontaneum*, and *Erianthus spp.*, 58 genotypes of relevance for the breeding program at Cenicaña differential response to the most crop-limiting pests and diseases in Colombia, 33 genotypes belonging to genetic introductions from other breeding programs around the world, commercial varieties in Colombia, and early selection stage genotypes from the breeding program at Cenicaña [88] (Additional file 1). In total, there were 189 modern genotypes, of which 60% were genotypes bred under Cenicaña’s breeding program (Additional file 1).

### Experimental design and data collection

The 220 genotypes were planted under an alpha-lattice design with 3 replications. This design belongs to an incomplete block design (IBD), which is used primarily in the reduction of the experimental error by splitting the total field variability in small incomplete blocks [90–93], minimizing the unknown variation within each replication [90, 91, 94, 95]. This design has been widely used in different crops such as rice [92], barley [91], wheat [91], and bread wheat [96], achieving great control of the experimental error. To increase the presicion in the control of the random errors, the commercial checks S29, S64, and S177 were replicated 8, 7, and 8 times, respectively, within each replicate block, resulting in 240 experimental units per replicate. In this study, each replication contained 12 blocks with 20 experimental units. The experimental unit was a plot of five rows, each 5 m long, with 1.65 m between rows. Agronomic practices were applied following commercial practices implemented by the sugarcane mill. The sampling unit consisted of two rows, the third and fourth row of each plot, to avoid border effects.

Data was collected on a plot basis for the accumulation of sucrose (%Cane) at 10 and 13 months after planting (map), considered from here on as early and normal maturity, respectively. For early maturity, measurements were taken from the plant cane and first ratoon, while for normal maturity, measurements were taken from the plant cane, first, and second ratoon. Sucrose (%Cane) was measured at early maturity using the CeniAD method [97], while the direct analysis (DAC) method [28] was employed at normal maturity. The CeniAD method consists of the evaluation of sucrose in three internodes, one from the apical, one from the middle, and one from the basal part of 11 mature stalks randomly selected from each genotype. On the other hand, the DAC method evaluates the sucrose content in 11 mature stalks by shredding the complete stalk (node and internode) from each genotype. For both methods, samples were shredded, and the juice was extracted with a hydraulic press. The quantification of sucrose was performed in the extracted juice with a near infrared (NIR) spectroscopy methodology.

### Data analysis

Data was analyzed by a combine analysis across crop cycle (i.e., plant cane, first ratoon, and second ratoon) [98]. Within this type of models, some factors get nested within the experimental unit, which becomes in block factors innate to the units of the experiment [98]. The statistical model used was as following:

$${Y}_{ijkl}= {C}_{i}+{R}_{j\left(i\right)}+{B}_{k\left(ij\right)}+{G}_{l }+{I}_{il}+{\epsilon }_{ijkl}$$

where ${Y}_{ijkl}$ corresponds to the sucrose content at early or normal maturity of the genotype $l$ planted in the incomplete block *k* nested in replication *j* of crop cycle *i*. Similarly, *C*_*i*_ corresponds to the effect of crop cycle $i$, *R*_*j*(*i*)_ to the replication $j$ nested within crop cycle $i$, ${B}_{k\left(ij\right)}$ to the incomplete block $k$ nested in replication $j$ and crop cycle $i$, ${G}_{l}$ to genotype $l$, ${I}_{ij}$ to the interaction between genotype $l$ and crop cycle *i*, and ${\epsilon }_{ijkl}$ to the random residual [98]. All effects were assumed to be random except for the crop cycle (${C}_{i}$). For this experiment, the genotypes comprise a random and representative selection for the sugarcane breeding pool used in Cenicaña (Cenicaña´s sugarcane diverse panel [88]) and for that they were assumed to be random effects. Outliers were detected by estimating the probability of obtaining a larger absolute value for each residual using a t-distribution [99, 100]. Subsequently, each p-value was adjusted with a Bonferroni correction at a 2% level of significance [101, 102]. After removing outliers, 16 models were evaluated by making all possible combinations between the random effects and by testing for homogeneity ($V\left({\epsilon }_{ijkl}\right)={\sigma }_{e}^{2}$) or heterogeneity ($V\left({\epsilon }_{ijkl}\right)={\sigma }_{e\left(i\right)}^{2}$) of the residual variance. To identify the best fitting model, the Bayesian Information Criterion (BIC) [103] was used. The Bayesian information criterium (BIC) evaluates models in terms of their posterior probabilities, penalizing the models based on the number of parameters it includes [104–106], with higher penalty for the models that includes a higher number of parameters [107]. Therefore, the lower the BIC value, the better the model balances the goodness of fit with parsimony (i.e., simplicity) [104–106]. With the selected model, the best linear unbiased predictors (BLUP) for the genotypes were obtained for further analysis [108]. Broad-sense heritability was calculated following Cullis heritability for unbalanced data, which takes into account the mean variance of the difference between two BLUPs and the genotypic variance [109]. All analyses were carried out using the Proc Mixed procedure of SAS (SAS Institute, Cary, NC).

### DNA extraction and sequencing

Genomic DNA was extracted from each of the 220 genotypes by following the phenol‒chloroform protocol [110]. The DNA concentration was determined with a Thermo Fisher ® Nanodrop 2000 spectrophotometer, while the integrity was verified using a 0.8% agarose gel. The genomic DNA was sequenced using Genotype-By-Sequencing (GBS) [111], Restriction-site Associated DNA Sequencing (RADSeq) [112], and Whole Genome Sequencing (WGS) strategies. DNA was digested with the restriction enzyme *Pst I* for the single-end GBS technique [111] and *Eco RI* for the paired-end RADseq technique [112]. DNA libraries, for both techniques, were constructed following sequencing service providers and the sequence process was carried out using an Illumina HiSeq 2000 system (Illumina, San Diego, California, USA). For WGS, DNA libraries were constructed following Novogene sequence provider (Novogene Bioinformatics Technology Co. Ltd). Briefly, the genomic DNA was randomly sheared into short fragments (~ 150 bp). Each fragment was end-repaired, A-tailed, and ligated with Illumina adapters. The fragments with adapters were PCR amplified, size selected, purified, and sequenced using a System Illumina NovaSeq platform (HWI-ST1276).

Raw reads quality was assessed for each strategy using cutadapt [113] and FastQC [114] by removing reads with a *Phred* score lower than 30. Cleaned data from each strategy were mapped to the CC 01-1940 monoploid reference genome [115] using bowtie 2.2.5 [116]. Genotyping and variant detection were performed using the “*MultisampleVariantsDetector*” of NGSEP 4.0.2 [117]. SNP calling was performed through the “*VCFFilter*” module of NGSEP v 4.0.2 [117], assuming a ploidy of 10, a minimum allelic frequency (i.e., MAF) of 1%, a calling rate of 75%, a minimum sequencing depth of 30X (at least 30 reads per position in the genome), a minimum genotyping quality of 30 on the *Phred* scale, a distance between markers of 1 bp, and keeping only biallelic markers. Finally, SNP markers called from GBS, RADSeq, and WGS were merged using SAMtools v.1.10 [118], leaving a total of 137,889 SNPs for each one of the 220 genotypes.

### Population structure, kinship, and GWAS analysis

The genetic structure for the 220-genotype population was estimated with a discriminant analysis of principal components (DAPC) with the adegenet package [119] implemented in R [120]. The optimal number of clusters or subpopulations was identified using the Bayesian Information Criterium [103]. The kinship matrix was calculated according to the VanRaden method [121], implemented in the statistical package GWASpoly 2.10 [122]. Genome-wide association analyses were performed with the GWASpoly 2.10 package [122]. Within this package, 12 genetic models were evaluated: general, additive, dominant simplex (1-dom-ref and 1-dom-alt), dominant duplex (2-dom-ref and 2-dom-alt), dominant triplex (3-dom-ref and 3-dom-alt), dominant tetraplex (4-dom-ref and 4-dom-alt), and dominant pentaplex (5-dom-ref and 5-dom-alt) [99]. Briefly, the general model allows each marker allele dosage to be arbitrary and statistically equivalent, while the additive model assumes that the effect of the marker is proportional to the dosage of the alternative allele [122]. For the dominant models, the presence of at least one, two, three, four, or five copies of the alternative (alt) or reference (ref) alleles generates the same effect as the homozygous genotype for this allele [122]. SNPs significantly associated with sucrose (%Cane) were identified using the false discovery rate (FDR) test with a significance level of 5% [123].

The presence of false positives in each model was visually determined through quantile‒quantile (QQ) plots, which assume a uniform distribution of p-values under the null hypothesis of no association [124–126]. For each genetic model, the most significant markers were progressively removed before reanalyzing the genetic association (GWAS). Then, a new QQ-plot was generated and deviations from the null hypothesis were analyzed. This process was repeated until there were only markers that did not deviate significantly from the null hypothesis. At this point, the remaining markers (i.e., those that fail to reject the null hypothesis) were identified as false positives and removed from the analysis [127]. Similarly, the proportion of phenotypic variance explained by each significant SNP (R^2^) was estimated using the Cox & Snell pseudo-R^2^ [128]. This R^2^ is based on the relationship between the likelihood of the null model (the intercept-only model) and the full model [129]. Finally, the linkage disequilibrium (LD) region was estimated at an average of 500 kb for all chromosomes (data not shown), and therefore, candidate genes were searched for in the region spanning 500 kb upstream and downstream of the marker. Once the genes were identified, they were filtered by selecting genes with known functions in plants. The function of each gene was manually evaluated, and only those involved in the production or accumulation of sucrose were selected.

### Electronic supplementary material

Below is the link to the electronic supplementary material.

Supplementary Material 1

Supplementary Material 2

## Data Availability

The datasets generated and/or analyzed during the current study are available in the repository https://github.com/fsilvaag/GWAS-Sugarcane-Sucrose.git.
