# Supplementary material for: Genetic association analysis in sugarcane (Saccharum spp.) for sucrose accumulation in humid environments in Colombia
Source: BMC Plant Biol. 2024 Jun 18;24:570. doi: 10.1186/s12870-024-05233-y (PMC11184777; doi:10.1186/s12870-024-05233-y)
Supplement: Supplementary file 2 — Supplementary Material 2 [file 12870_2024_5233_MOESM2_ESM.pdf]

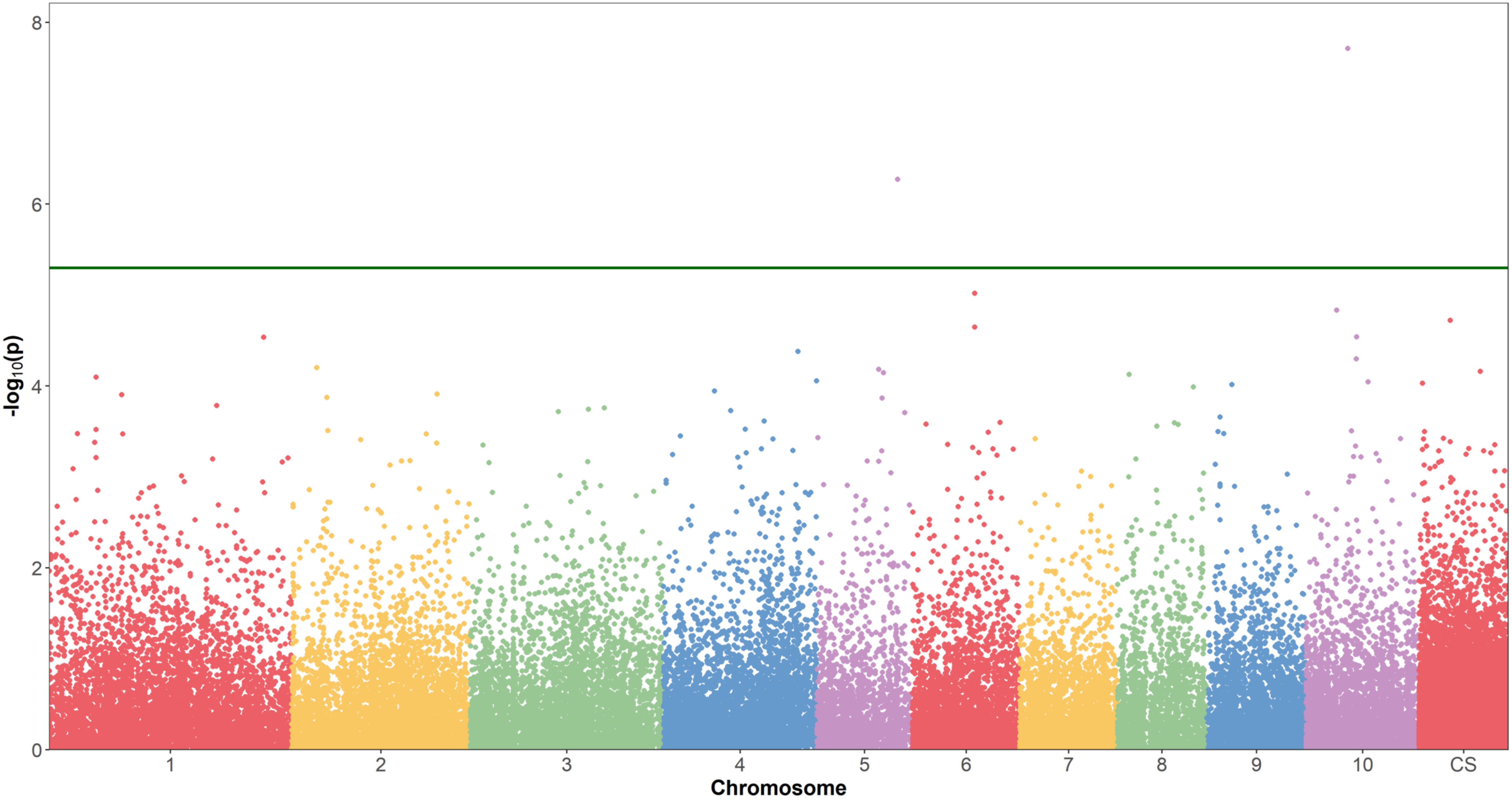

Figure S1. Manhattan plots for the accumulation of sucrose at early maturity (10 months after planting) along the monoploid genome of the variety CC 01-1940 (chromosomes numbered 1 to 10, CS indicating contigs and scaffolds) in the 1-dom-alt genetic model. The green line indicates the genome-wide threshold of p = 1x10-5.

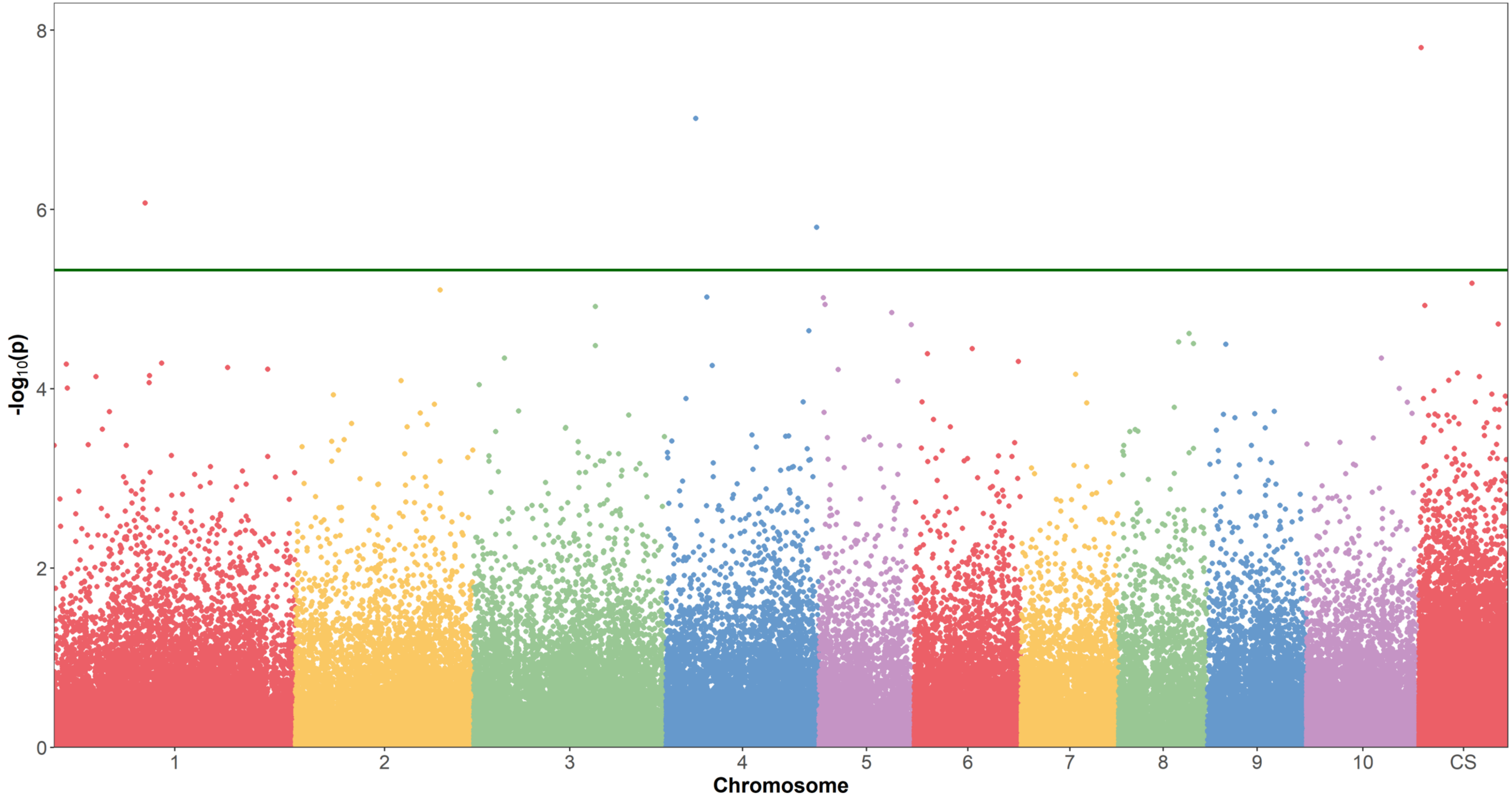

Figure S2. Manhattan plots for the accumulation of sucrose at early maturity (10 months after planting) along the monoploid genome of the variety CC 01-1940 (chromosomes numbered 1 to 10, CS indicating contigs and scaffolds) in the 1-dom-ref genetic model. The green line indicates the genome-wide threshold of p = 1x10-5.

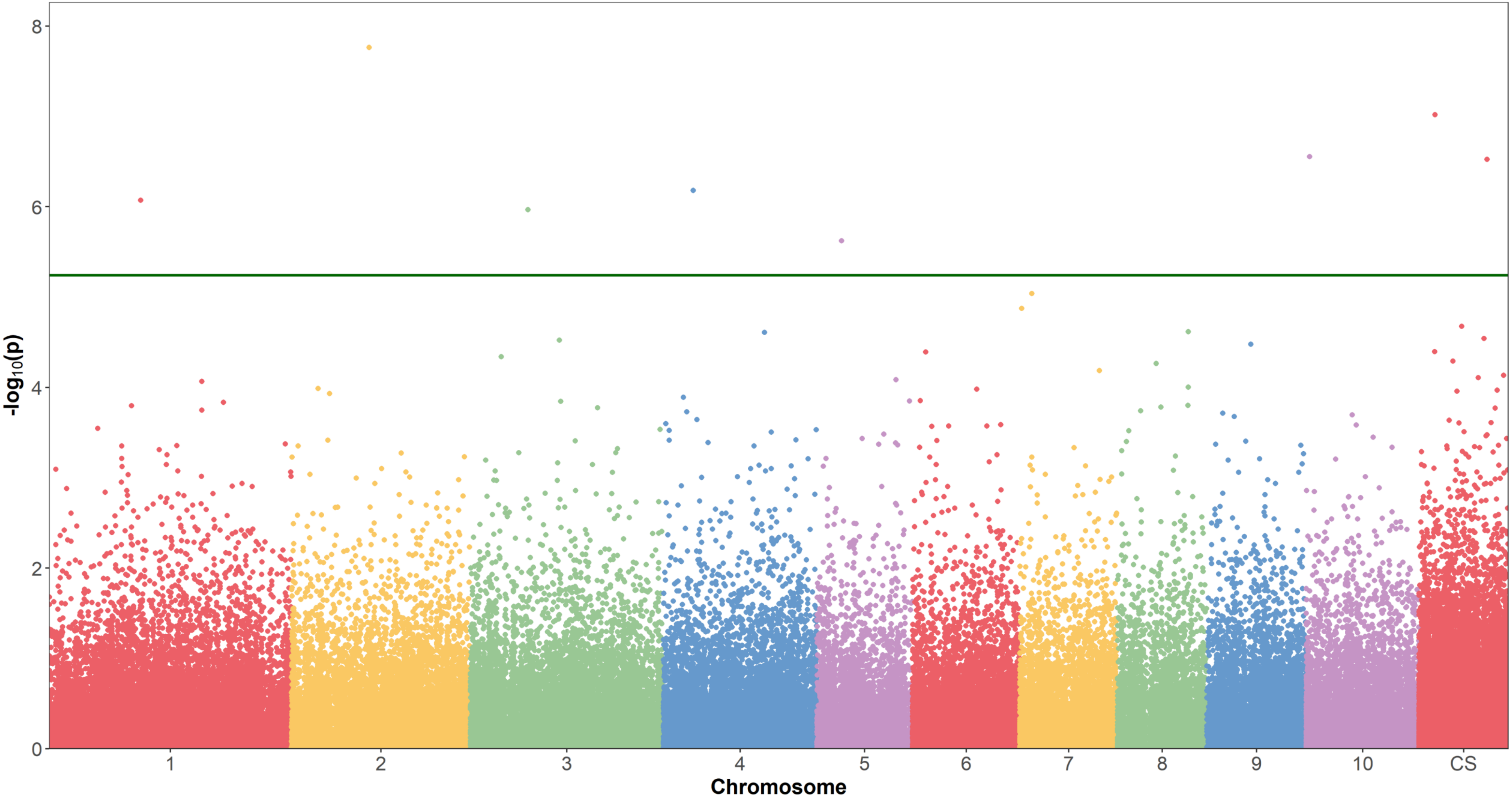

Figure S3. Manhattan plots for the accumulation of sucrose at early maturity (10 months after planting) along the monoploid genome of the variety CC 01-1940 (chromosomes numbered 1 to 10, CS indicating contigs and scaffolds) in the 2-dom-ref genetic model. The green line indicates the genome-wide threshold of p = 1x10-5.

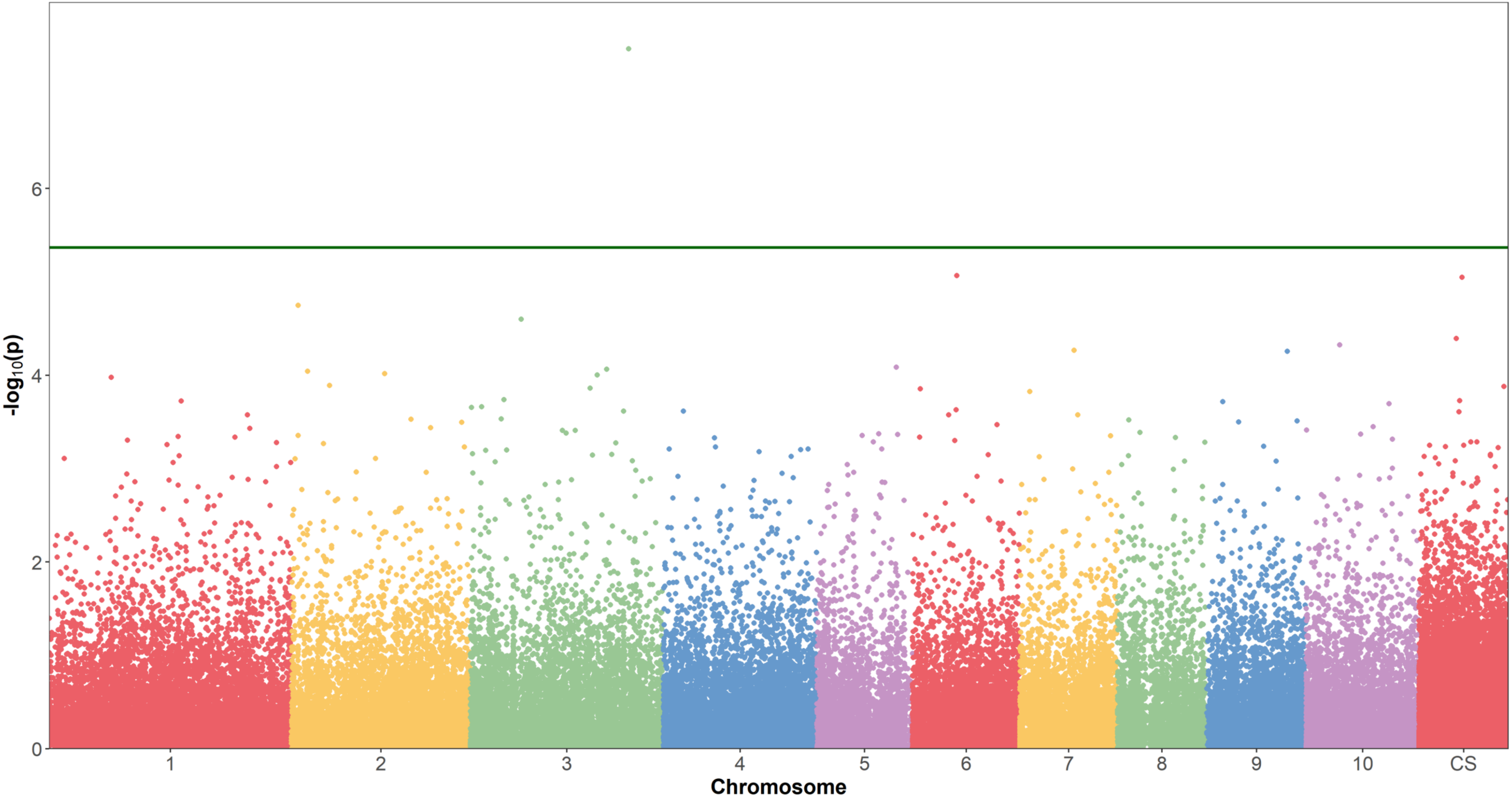

Figure S4. Manhattan plots for the accumulation of sucrose at early maturity (10 months after planting) along the monoploid genome of the variety CC 01-1940 (chromosomes numbered 1 to 10, CS indicating contigs and scaffolds) in the 4-dom-alt genetic model. The green line indicates the genome-wide threshold of p = 1x10-5.

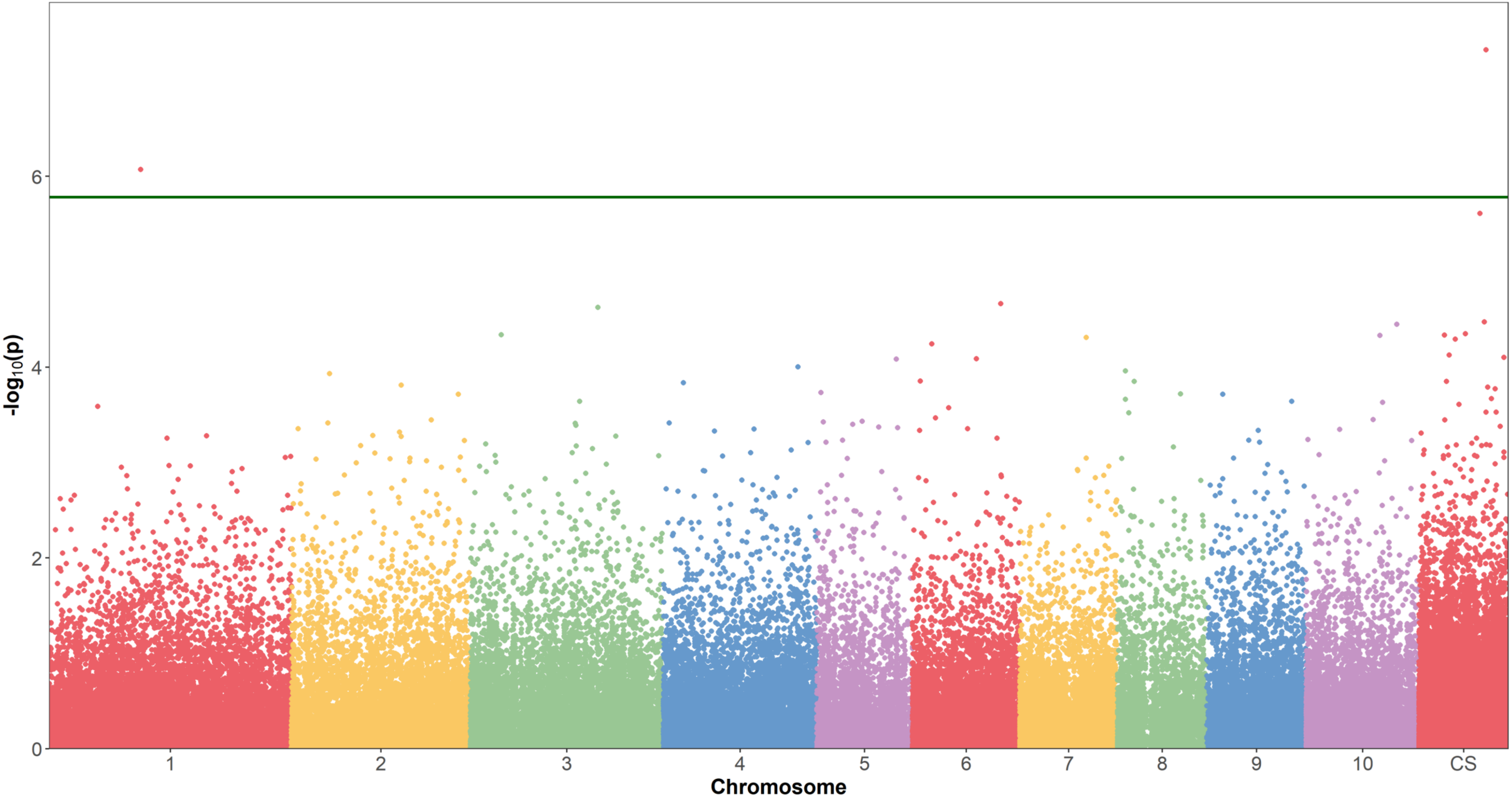

Figure S5. Manhattan plots for the accumulation of sucrose at early maturity (10 months after planting) along the monoploid genome of the variety CC 01-1940 (chromosomes numbered 1 to 10, CS indicating contigs and scaffolds) in the 4-dom-ref genetic model. The green line indicates the genome-wide threshold of p = 1x10-5.

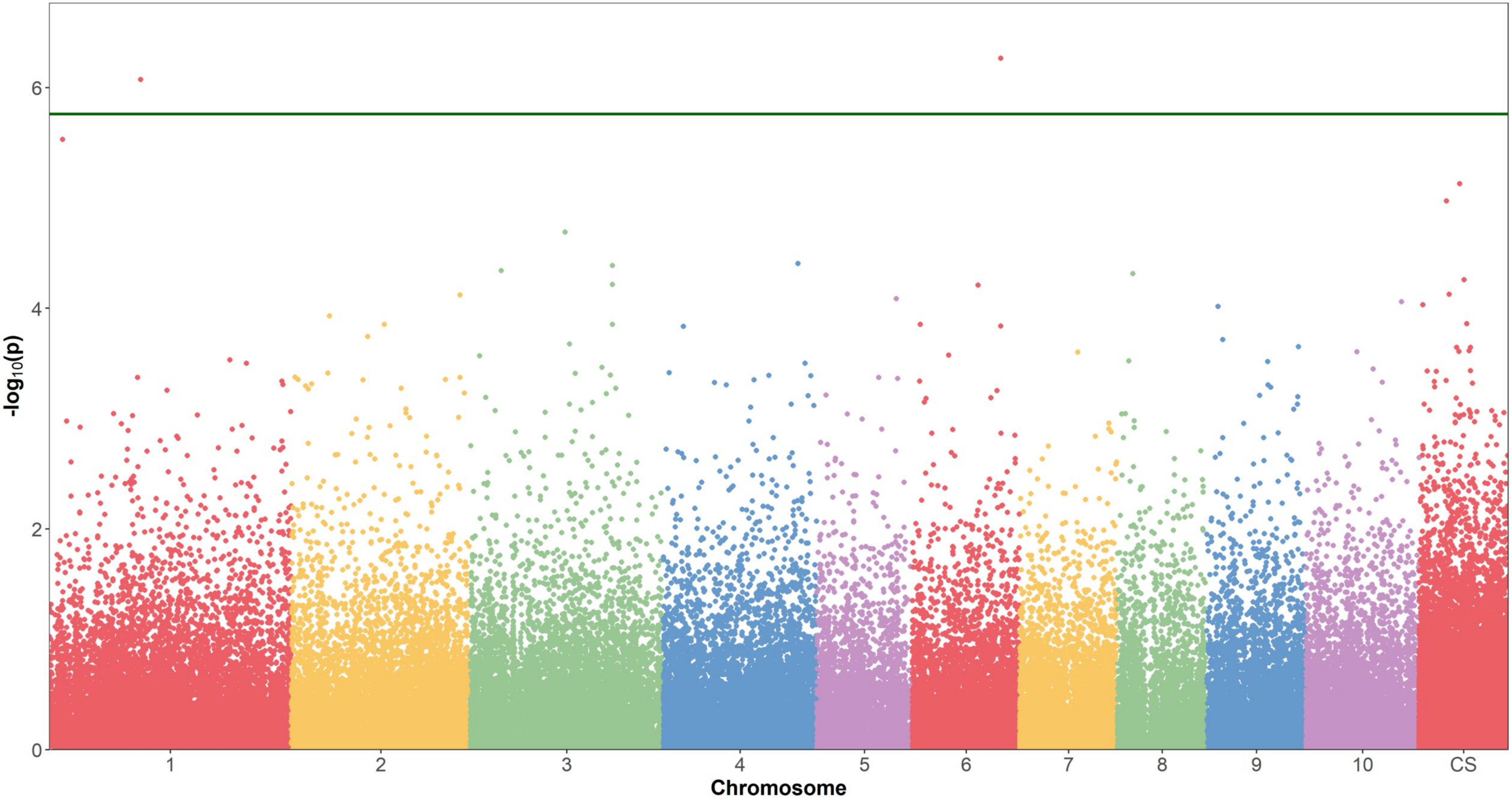

Figure S6. Manhattan plots for the accumulation of sucrose at early maturity (10 months after planting) along the monoploid genome of the variety CC 01-1940 (chromosomes numbered 1 to 10, CS indicating contigs and scaffolds) in the 5-dom-ref genetic model. The green line indicates the genome-wide threshold of p = 1x10-5.

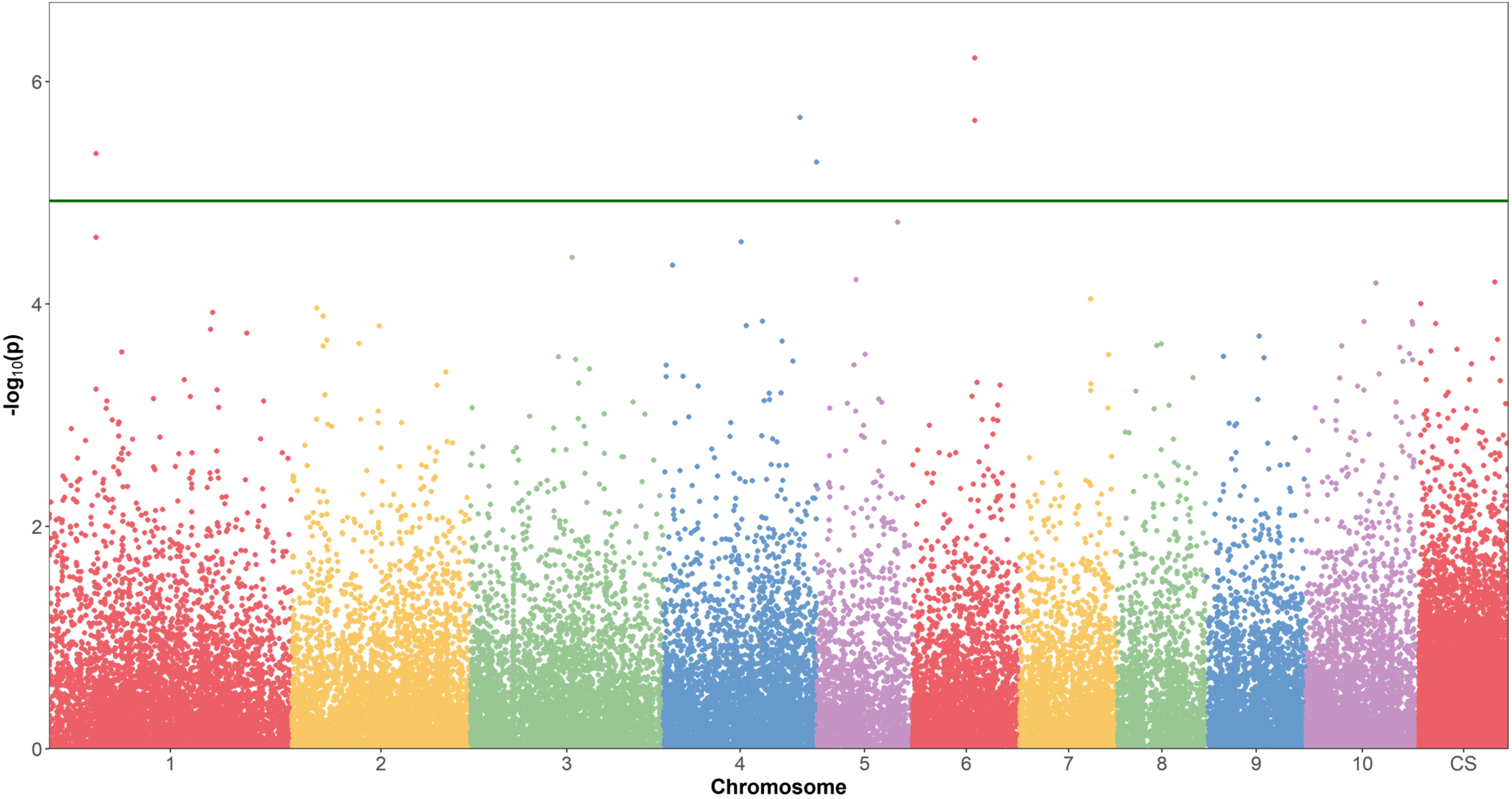

Figure S7. Manhattan plots for the accumulation of sucrose at normal maturity (13 months after planting) along the monoploid genome of the variety CC 01-1940 (chromosomes numbered 1 to 10, CS indicating contigs and scaffolds) in the 1-dom-alt genetic model. The green line indicates the genome-wide threshold of p = 1x10-5.

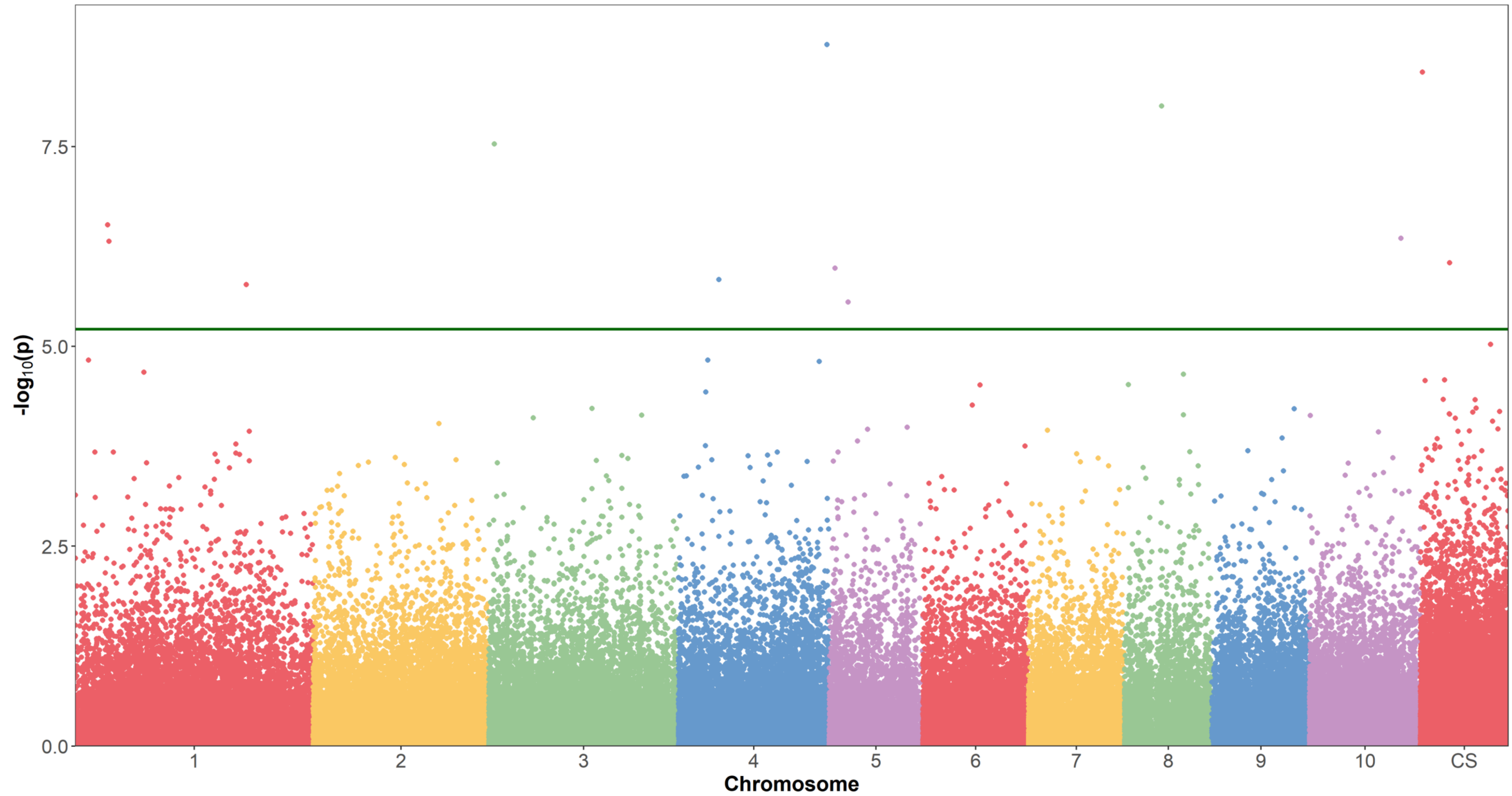

Figure S8. Manhattan plots for the accumulation of sucrose at normal maturity (13 months after planting) along the monoploid genome of the variety CC 01-1940 (chromosomes numbered 1 to 10, CS indicating contigs and scaffolds) in the 1-dom-ref genetic model. The green line indicates the genome-wide threshold of p = 1x10-5.

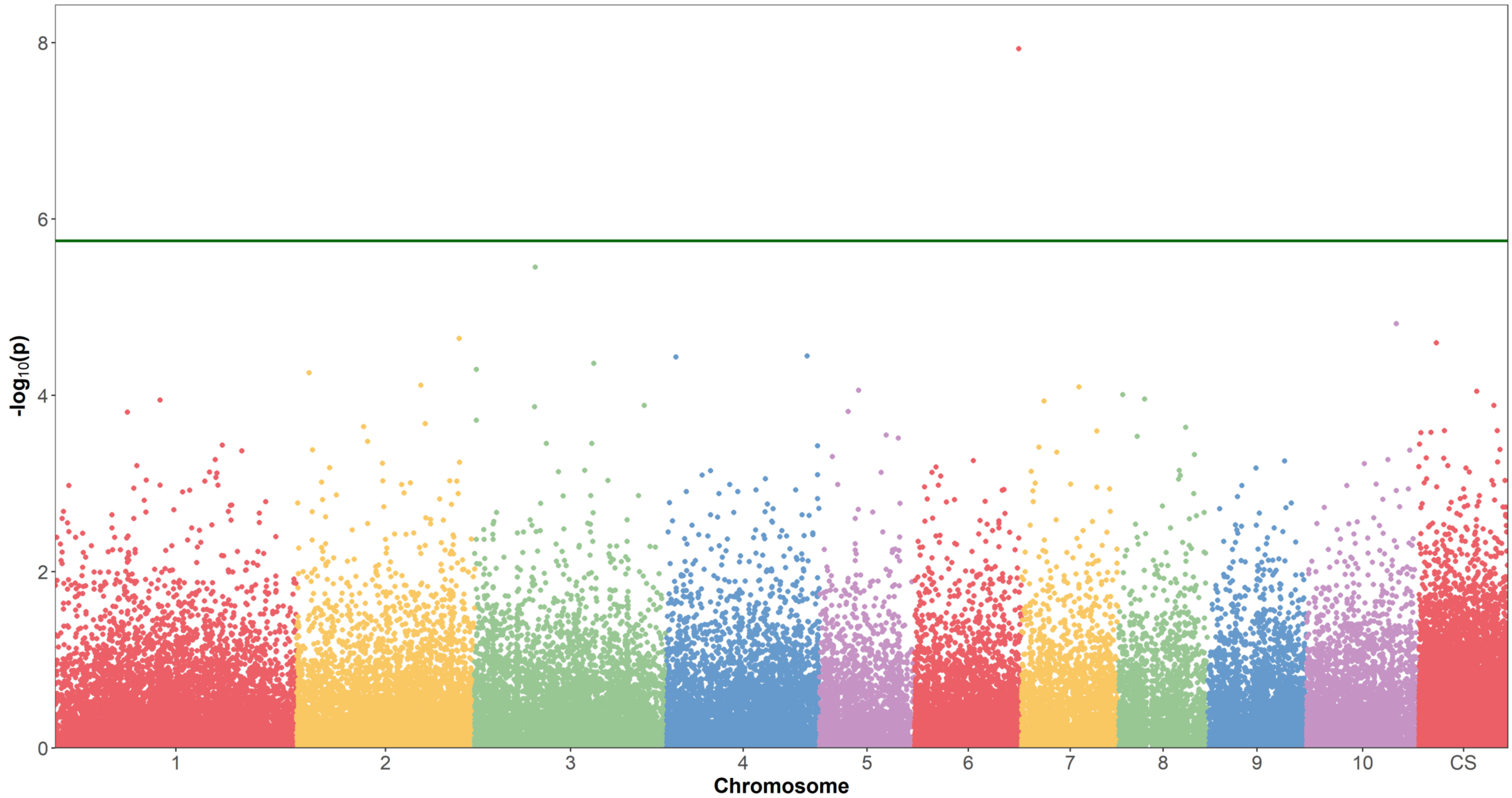

Figure S9. Manhattan plots for the accumulation of sucrose at normal maturity (13 months after planting) along the monoploid genome of the variety CC 01-1940 (chromosomes numbered 1 to 10, CS indicating contigs and scaffolds) in the 2-dom-alt genetic model. The green line indicates the genome-wide threshold of p = 1x10-5.

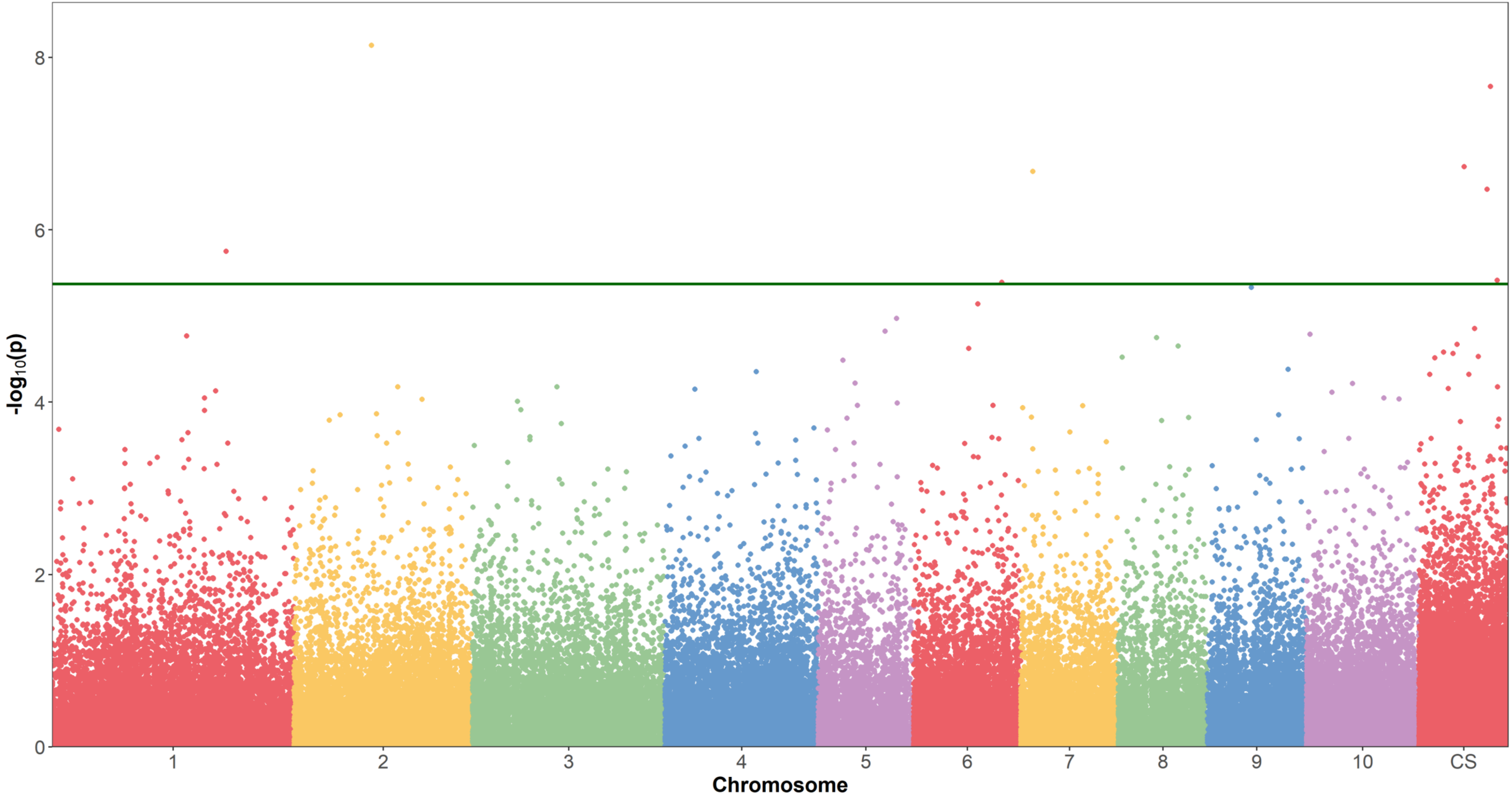

Figure S10. Manhattan plots for the accumulation of sucrose at normal maturity (13 months after planting) along the monoploid genome of the variety CC 01-1940 (chromosomes numbered 1 to 10, CS indicating contigs and scaffolds) in the 2-dom-ref genetic model. The green line indicates the genome-wide threshold of p = 1x10-5.

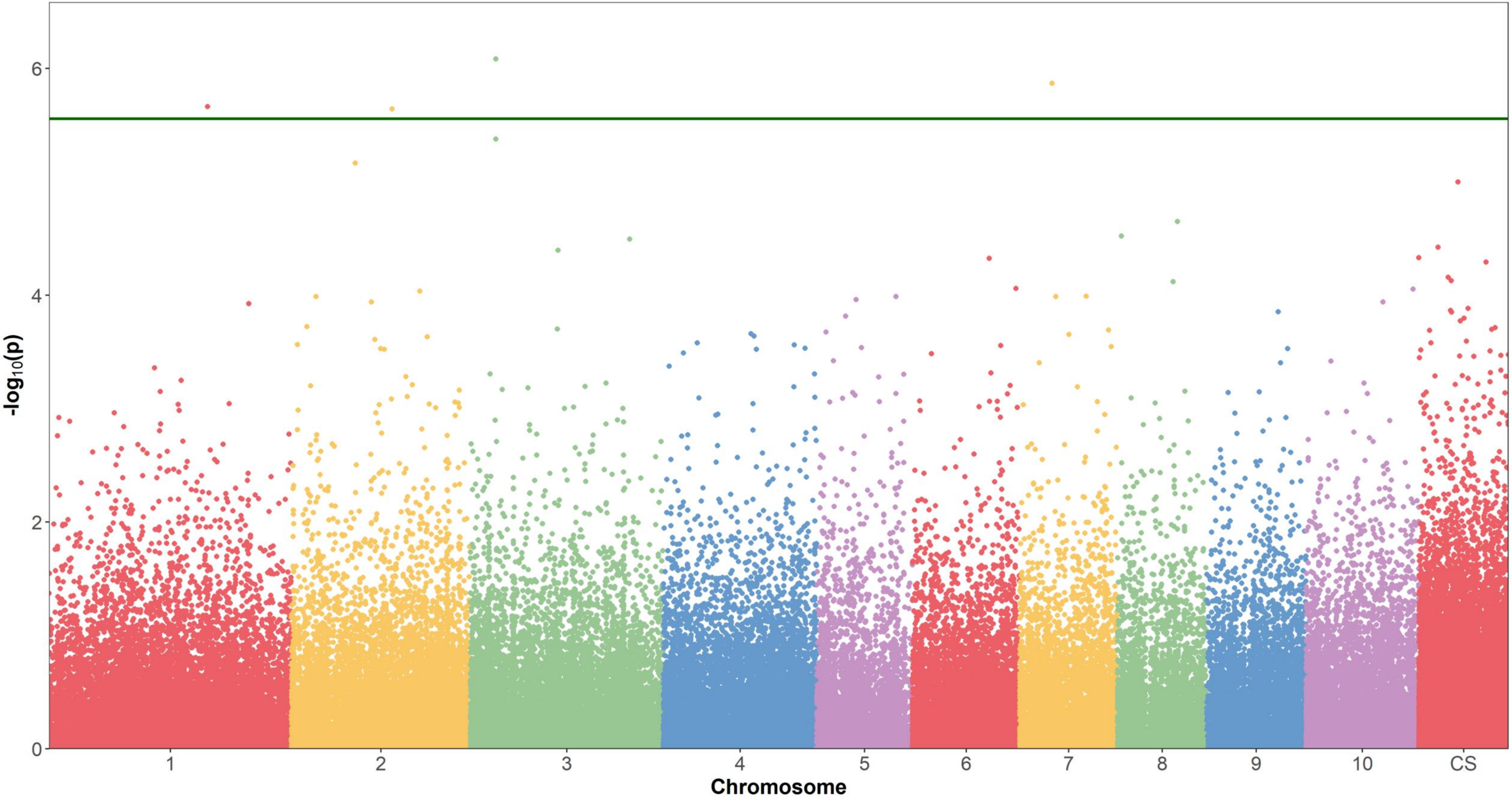

Figure S11. Manhattan plots for the accumulation of sucrose at normal maturity (13 months after planting) along the monoploid genome of the variety CC 01-1940 (chromosomes numbered 1 to 10, CS indicating contigs and scaffolds) in the 3-dom-ref genetic model. The green line indicates the genome-wide threshold of p = 1x10-5.

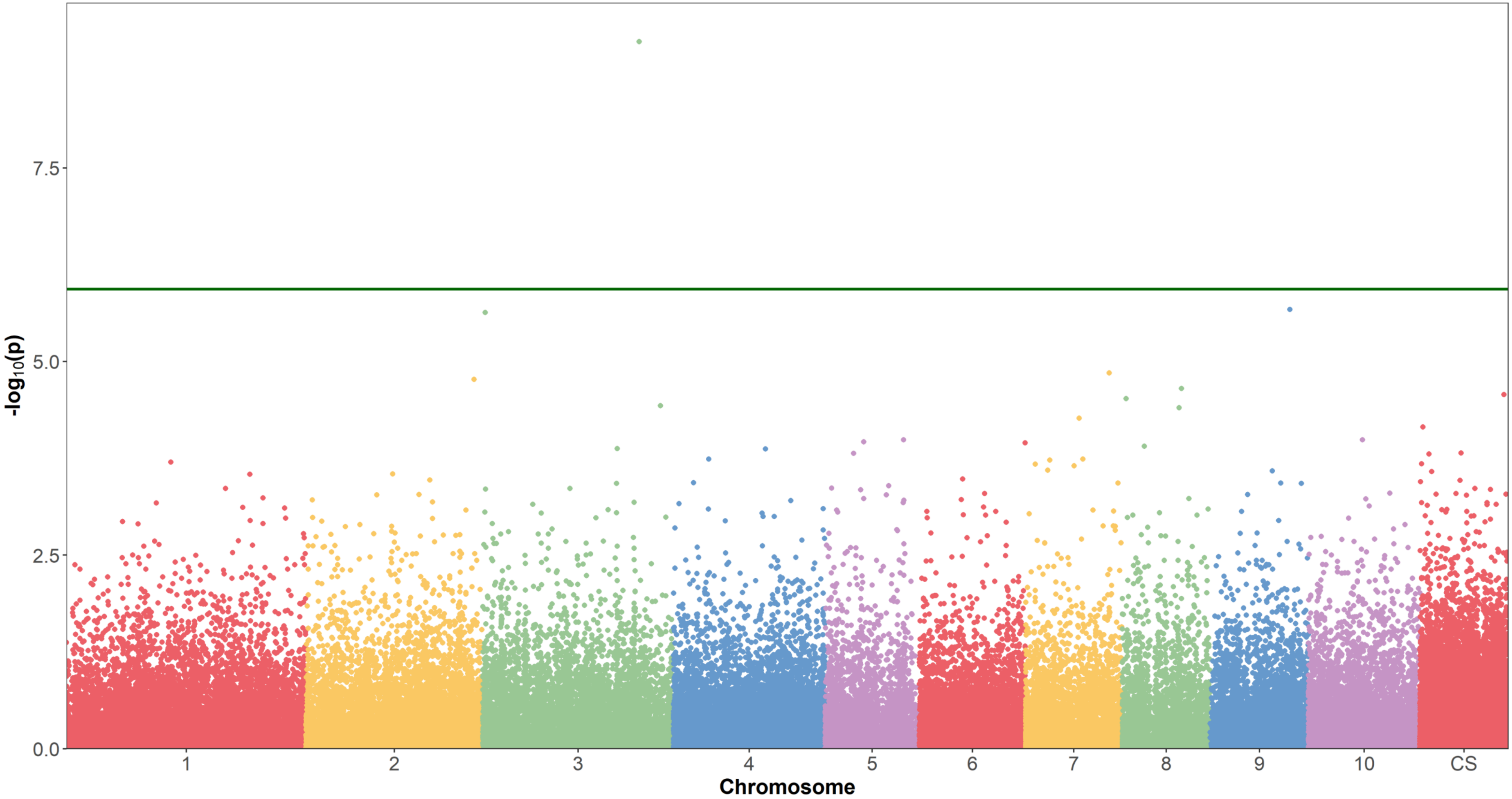

Figure S12. Manhattan plots for the accumulation of sucrose at normal maturity (13 months after planting) along the monoploid genome of the variety CC 01-1940 (chromosomes numbered 1 to 10, CS indicating contigs and scaffolds) in the 4-dom-alt genetic model. The green line indicates the genome-wide threshold of p = 1x10-5.

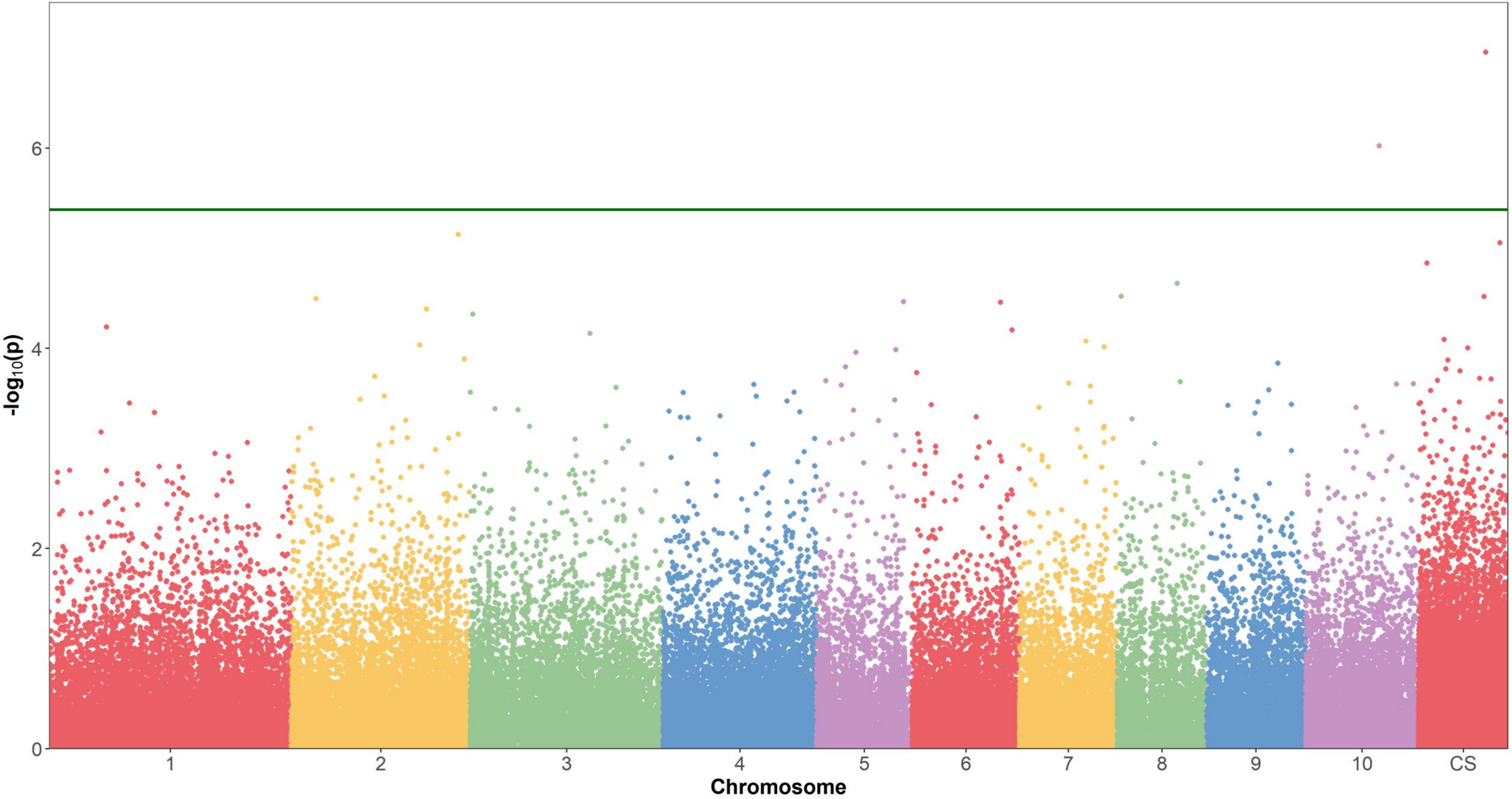

Figure S13. Manhattan plots for the accumulation of sucrose at normal maturity (13 months after planting) along the monoploid genome of the variety CC 01-1940 (chromosomes numbered 1 to 10, CS indicating contigs and scaffolds) in the 4-dom-ref genetic model. The green line indicates the genome-wide threshold of p = 1x10-5.

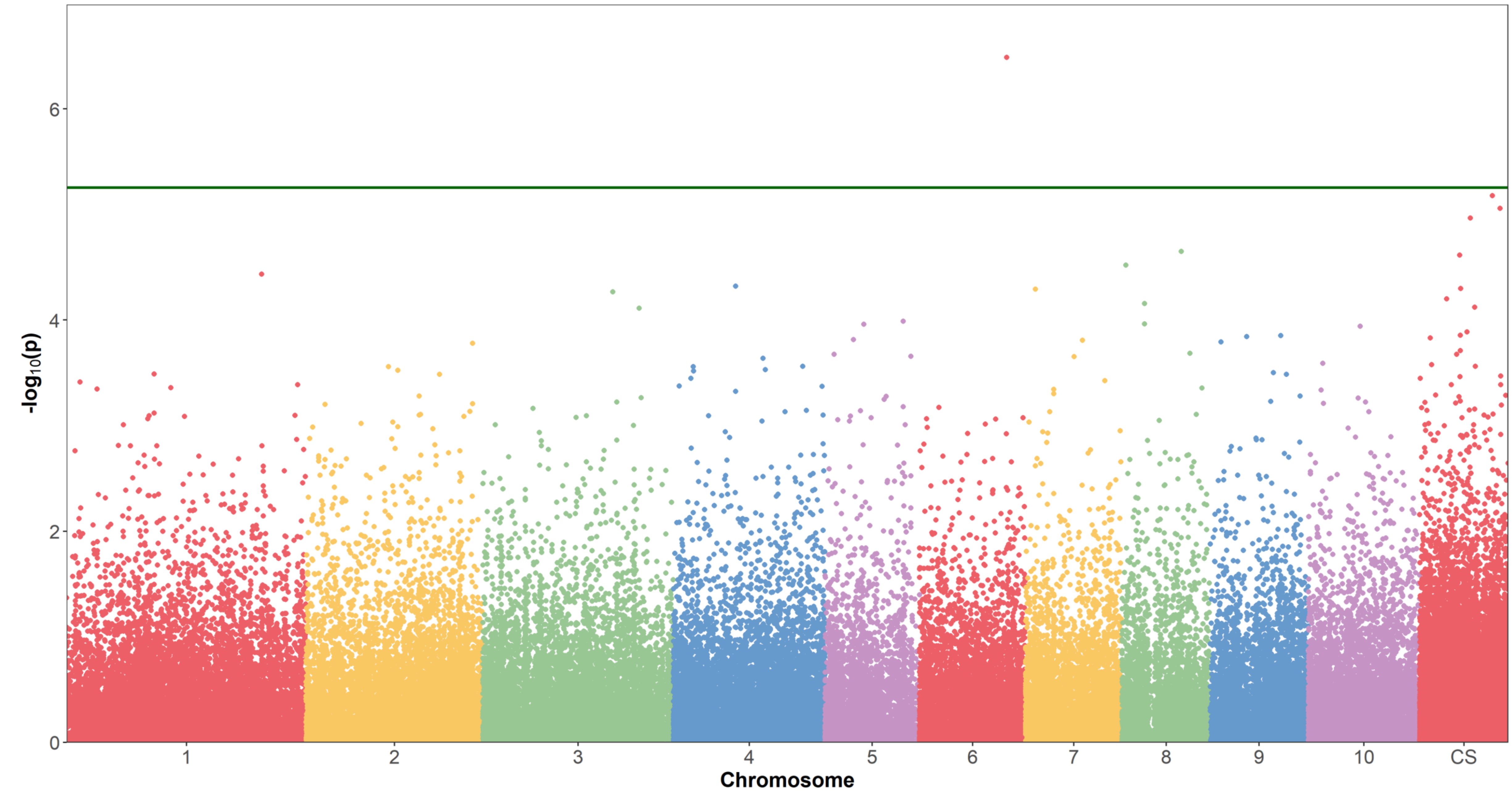

Figure S14. Manhattan plots for the accumulation of sucrose at normal maturity (13 months after planting) along the monoploid genome of the variety CC 01-1940 (chromosomes numbered 1 to 10, CS indicating contigs and scaffolds) in the 5-dom-ref genetic model. The green line indicates the genome-wide threshold of p = 1x10-5.
